# Receipt of Selected Preventive Health Services for Women and Men of Reproductive Age — United States, 2011–2013

**DOI:** 10.15585/mmwr.ss6620a1

**Published:** 2017-10-27

**Authors:** Karen Pazol, Cheryl L. Robbins, Lindsey I. Black, Katherine A. Ahrens, Kimberly Daniels, Anjani Chandra, Anjel Vahratian, Lorrie E. Gavin

**Affiliations:** 1 Division of Reproductive Health, National Center for Chronic Disease Prevention and Health Promotion, CDC, Atlanta, Georgia; 2 Division of Health Interview Statistics, National Center for Health Statistics, CDC, Hyattsville, Maryland; 3 Office of Population Affairs, U.S. Department of Health and Human Services, Rockville, Maryland; 4 Division of Vital Statistics, National Center for Health Statistics, CDC, Hyattsville, Maryland

## Abstract

**Problem/Condition:**

Receipt of key preventive health services among women and men of reproductive age (i.e., 15–44 years) can help them achieve their desired number and spacing of healthy children and improve their overall health. The 2014 publication *Providing Quality Family Planning Services: Recommendations of CDC and the U.S. Office of Population Affairs* (QFP) establishes standards for providing a core set of preventive services to promote these goals. These services include contraceptive care for persons seeking to prevent or delay pregnancy, pregnancy testing and counseling, basic infertility services for those seeking to achieve pregnancy, sexually transmitted disease (STD) services, and other preconception care and related preventive health services. QFP describes how to provide these services and recommends using family planning and other primary care visits to screen for and offer the full range of these services. This report presents baseline estimates of the use of these preventive services before the publication of QFP that can be used to monitor progress toward improving the quality of preventive care received by women and men of reproductive age.

**Period Covered:**

2011–2013.

**Description of the System:**

Three surveillance systems were used to document receipt of preventive health services among women and men of reproductive age as recommended in QFP. The National Survey of Family Growth (NSFG) collects data on factors that influence reproductive health in the United States since 1973, with a focus on fertility, sexual activity, contraceptive use, reproductive health care, family formation, child care, and related topics. NSFG uses a stratified, multistage probability sample to produce nationally representative estimates for the U.S. household population of women and men aged 15–44 years. This report uses data from the 2011–2013 NSFG.

The Pregnancy Risk Assessment Monitoring System (PRAMS) is an ongoing, state- and population-based surveillance system designed to monitor selected maternal behaviors and experiences that occur before, during, and shortly after pregnancy among women who deliver live-born infants in the United States. Annual PRAMS data sets are created and used to produce statewide estimates of preconception and perinatal health behaviors and experiences. This report uses PRAMS data for 2011–2012 from 11 states (Hawaii, Maine, Maryland, Michigan, Minnesota, Nebraska, New Jersey, Tennessee, Utah, Vermont, and West Virginia).

The National Health Interview Survey (NHIS) is a nationally representative survey of noninstitutionalized civilians in the United States. NHIS collects data on a broad range of health topics, including the prevalence, distribution, and effects of illness and disability and the services rendered for or because of such conditions. Households are identified through a multistage probability household sampling design, and estimates are produced using weights that account for the sampling design, nonresponse, and poststratification adjustments. This report uses data from the 2013 NHIS for women aged 18–44 years.

**Results:**

Many preventive health services recommended in QFP were not received by all women and men of reproductive age. For contraceptive services, including contraceptive counseling and advice, 46.5% of women aged 15–44 years at risk for unintended pregnancy received services in the past year, and 4.5% of men who had vaginal intercourse in the past year received services in that year. For sexually transmitted disease (STD) services, among all women aged 15–24 years who had oral, anal, or vaginal sex with an opposite sex partner in the past year, 37.5% were tested for chlamydia in that year. Among persons aged 15–44 years who were at risk because they were not in a mutually monogamous relationship during the past year, 45.3% of women were tested for chlamydia and 32.5% of men were tested for any STD in that year. For preconception care and related preventive health services, data from selected states indicated that 33.2% of women with a recent live birth (i.e., 2–9 months postpartum) talked with a health care professional about improving their health before their most recent pregnancy; of selected preconception counseling topics, the most frequently discussed was taking vitamins with folic acid before pregnancy (81.2%), followed by achieving a healthy weight before pregnancy (62.9%) and how drinking alcohol (60.3%) or smoking (58.2%) during pregnancy can affect a baby. Nationally, among women aged 18–44 years irrespective of pregnancy status, 80.9% had their blood pressure checked by a health care professional and 31.7% received an influenza vaccine in the past year; 54.5% of those with high blood pressure were tested for diabetes, 44.9% of those with obesity had a health care professional talk with them about their diet, and 55.2% of those who were current smokers had a health professional talk with them about their smoking in the past year. Among all women aged 21–44 years, 81.6% received a Papanicolaou (Pap) test in the past 3 years.

Receipt of certain preventive services varied by age and race/ethnicity. Among women with a recent live birth, the percentage of those who talked with a health care professional about improving their health before their most recent pregnancy increased with age (range: 25.9% and 25.2% for women aged ≤19 and 20–24 years, respectively, to 35.9% and 37.8% for women aged 25–34 and ≥35 years, respectively). Among women with a recent live birth, the percentage of those who talked with a health care professional about improving their health before their most recent pregnancy was higher for non-Hispanic white (white) (35.2%) compared with non-Hispanic black (black) (30.0%) and Hispanic (26.0%) women. Conversely, across most STD screening services evaluated, testing was highest among black women and men and lowest among their white counterparts.

Receipt of many preventive services recommended in QFP increased consistently across categories of family income and continuity of health insurance coverage. Prevalence of service receipt was highest among women in the highest family income category (>400% of federal poverty level [FPL]) and among women with insurance coverage for each of the following: contraceptive services among women at risk for unintended pregnancy; medical services beyond advice to help achieve pregnancy; vaccinations (hepatitis B and human papillomavirus [HPV], ever; tetanus, past 10 years; influenza, past year); discussions with a health care professional about improving health before pregnancy and taking vitamins with folic acid; blood pressure and diabetes screening; discussions with a health care professional in the past year about diet, among those with obesity; discussions with a health care professional in the past year about smoking, among current smokers; Pap tests within the past 3 years; and mammograms within the past 2 years.

**Interpretation:**

Before 2014, many women and men of reproductive age were not receiving several of the preventive services recommended for them in QFP. Although differences existed by age and race/ethnicity, across the range of recommended services, receipt was consistently lower among women and men with lower family income and greater instability in health insurance coverage.

**Public Health Action:**

Information in this report on baseline receipt during 2011–2013 of preventive services for women and men of reproductive age can be used to target improvements in the use of recommended services through the development ofresearch priorities, information for decision makers, and public health practice. Health care administrators and practitioners can use the information to identify subpopulations with the greatest need for preventive services and make informed decisions on resource allocation. Public health researchers can use the information to guide research on the determinants of service use and factors that might increase use of preventive services. Policymakers can use this information to evaluate the impact of policy changes and assess resource needs for effective programs, research, and surveillance on the use of preventive health services for women and men of reproductive age.

## Introduction

In 2014, CDC published *Providing Quality Family Planning Services: Recommendations of CDC and the U.S. Office of Population Affairs* (QFP) (*1*,*2*). QFP recommends a core set of preventive health services for women and men of reproductive age (i.e., 15–44 years) that can help them achieve their desired number and spacing of healthy children and improve their overall health. Recommended services include contraceptive care for persons seeking to prevent or delay pregnancy, pregnancy testing and counseling, basic infertility services for those seeking to achieve pregnancy, sexually transmitted disease (STD) services, and other preconception care and related preventive health services (e.g., screening for smoking, obesity, diabetes, high blood pressure, and breast and cervical cancer). QFP encourages use of the family planning visit to assess the need for each of these services and offer them as recommended. QFP also encourages screening all women and men of reproductive age about their need for family planning services, even when their initial reason for seeking services is not related to preventing or achieving pregnancy. For each of these preventive services, QFP describes the subpopulations recommended to receive them and how often they should be provided (Appendix A).

These family planning services are recommended because of evidence of their protective effect on adverse pregnancy outcomes and other conditions that affect the overall health of women and men of reproductive age (*1*–*5*). Illustrating the need for these services, the most recent estimates for the United States indicate that each year 2.8 million (45%) of all pregnancies are unintended (*6*), approximately 30% of births occur within an interpregnancy interval <18 months since the last live birth (*7*), and approximately 9.6% of infants are born preterm (*8*). In addition, many women of reproductive age have health conditions (e.g., obesity, hypertension, diabetes, and STDs) that can adversely affect their health and the health of their future pregnancies (*9*–*11*) (Appendix B), and 6.7% of married women of reproductive age are infertile (*12*). The hypothesized pathway between delivery of recommended preventive services and improved health outcomes illustrates that recommendations and implementation support lead to receipt of preventive services (short-term outcomes), which contributes to improvements in health-related behaviors and other intermediate outcomes that result in improvements in targeted health conditions (long-term health outcomes) (Figure).

**FIGURE:**
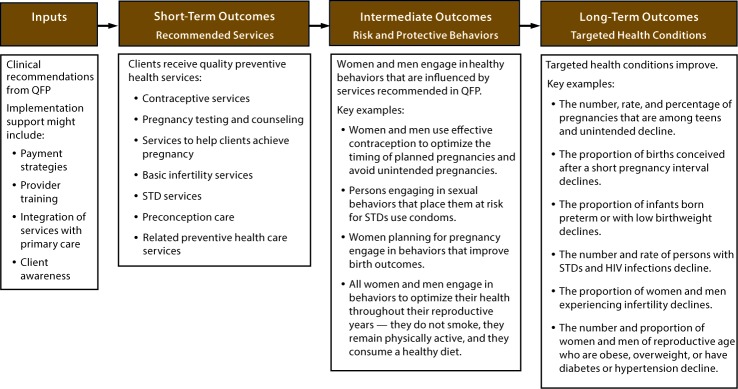
Pathway between delivery of preventive health services recommended in *Providing Quality Family Planning Services: Recommendations of CDC and the U.S. Office of Population Affairs* and improved health outcomes **Abbreviations:** HIV = human immunodeficiency virus; QFP = *Providing Quality Family Planning Services: Recommendations of CDC and the U.S. Office of Population Affairs*; STD = sexually transmitted disease.

Provision of the preventive services recommended in QFP is also cost-effective and can result in substantial cost savings. For example, a recent analysis of 28 clinical preventive services, including many recommended in QFP (e.g., screening for tobacco and alcohol use, cervical cancer, hypertension, obesity, and human immunodeficiency virus [HIV] and STDs such as chlamydia and gonorrhea), illustrates the cost-effectiveness of these services (*13*). Further, in 2010, the $2.2 billion investment in public funding for family planning programs and providers saved $15.8 billion, or approximately $7 for every $1 spent, by averting the need for public expenditures related to cervical cancer, HIV and other sexually transmitted infections, infertility, unintended pregnancy, and low birth weight and preterm births (*14*).

The services recommended in QFP were developed on the basis of a rigorous review process (*1*,*2*). Whenever possible, QFP followed existing clinical recommendations from CDC, the U.S. Preventive Services Task Force (USPSTF), and professional medical organizations such as the American College of Obstetricians and Gynecologists and the American Academy of Pediatrics (*15**–**28*); yet QFP also fills gaps in existing clinical recommendations, particularly with regard to provision of contraceptive counseling.

This report presents estimates of the use of recommended preventive health services among women and men aged 15–44 years during 2011–2013, the period shortly before QFP was published. By aligning the subpopulation included in each estimate with the population recommended for receipt of each service and matching the timeframe with the recommended interval for each service, this report documents the baseline receipt of preventive health services among persons in need of them, before the publication of QFP. The findings can be used to identify populations that were not receiving recommended preventive health services and opportunities for improving access, particularly where notable gaps exist.

## Methods

### Description of the Surveillance Systems

The National Survey of Family Growth (NSFG) is conducted by CDC’s National Center for Health Statistics in collaboration with other agencies of the U.S. Department of Health and Human Services. NSFG focuses on fertility, sexual activity, contraceptive use, reproductive health care, family formation, child care, and other topics among the U.S. household population of women and men of reproductive age. After six periodic cycles from 1973 to 2002, NSFG has employed a continuous fieldwork design since 2006, with interviews conducted over 48 weeks every year. NSFG uses a stratified, multistage probability sample to create nationally representative estimates of sexual behavior, contraceptive use, and sexual and reproductive health care service usage. Details of NSFG have been described elsewhere (*29*) and are available at https://www.cdc.gov/nchs/nsfg/index.htm. This report uses data from the 2011–2013 NSFG, which had response rates of 73.4% and 72.1% for a final sample size of 5,601 women and 4,815 men, respectively.

The Pregnancy Risk Assessment Monitoring System (PRAMS) is an ongoing, state- and population-based surveillance system designed to monitor selected self-reported maternal behaviors and experiences that occur before, during, and after pregnancy among women who deliver a live-born infant in participating U.S. states and New York City. All PRAMS reporting areas use a standardized data collection method developed by CDC. This standard method uses a mixed mode delivery, including mailed questionnaires beginning 2–3 months after the delivery of a live-born infant to allow for collection of information related to postpartum maternal and infant experiences and up to 15 follow-up telephone calls with nonresponders during the first 9 months of the postpartum period. The questionnaire consists of a core set of questions included for all reporting areas and standard questions chosen by each reporting area from a pretested list. Survey data are linked to selected birth certificate data and weighted to account for complex sample design, nonresponse, and noncoverage. Details of PRAMS methodology have been described previously (*30*) and are available at https://www.cdc.gov/PRAMS/. This report uses 2011–2012 PRAMS data from 11 states (Hawaii, Maine, Maryland, Michigan, Minnesota, Nebraska, New Jersey, Tennessee, Utah, Vermont, and West Virginia) that achieved an overall weighted response rate of 65%. The data included in this report are from responses to standard questions adopted selectively by individual states, and thus the number of states and women included in each estimate varies by question.

The National Health Interview Survey (NHIS), conducted by the National Center for Health Statistics, is an annual, nationally representative, in-person survey and is one of the nation’s primary sources of general health information. Data from NHIS provide annual estimates of health care access and use, health conditions, health behaviors, and other health-related information for the civilian noninstitutionalized U.S. population. Interviews are conducted in respondents’ homes. In some instances, follow-up telephone calls are conducted to complete the interviews. Selected health and demographic information is collected for all household members; one sample child (if any children aged ≤17 years are present) and one sample adult are randomly selected from each family in NHIS to answer more detailed health-related questions. Although some overlap in content exists, the sample child and sample adult respondents answer different questions; therefore, only estimates for sample adult women aged 18–44 years are within the scope of this report. Details of NHIS have been described elsewhere (*31*) and are available at https://www.cdc.gov/nchs/nhis/index.htm. This report uses data from the 2013 NHIS, which had a response rate of 61.2% for the sample adult component for a final sample size of 8,244 women aged 18–44 years.

### Selection of Indicators

Indicators were selected for each of the service areas recommended in QFP for women of reproductive age and select services recommended for men of reproductive age. Estimates for use of services related to preventing, delaying, or achieving pregnancy (contraception and medical services for achieving pregnancy) were obtained from NSFG. Estimates for STD prevention services (STD testing and vaccines) were obtained from NSFG and NHIS. Estimates for use of preconception care services among women with a recent live birth were obtained from PRAMS. Estimates of preconception care and related preventive health services among women, irrespective of pregnancy status, were obtained from NHIS.

In defining each service variable, the timeframe was matched to the interval recommended in QFP for receipt of that service, and the subpopulation in need of each service was aligned as closely as possible to the subpopulation recommended in QFP to receive that service. Because of this alignment, for the majority of the services in this report, receipt among the full included population should be considered the standard against which estimates can be compared. However, for some clinical recommendations, the frequency of screening is not specified (e.g., some USPSTF recommendations encourage periodic screening without definition of the term), which should be considered when interpreting these findings. For other services, estimates were generated across strata to separate out populations where less complete usage might be expected. For example, QFP recommends that contraceptive services, including contraceptive counseling,* be offered to women who are at risk for unintended pregnancy.^†^Accordingly, only women who were at risk for unintended pregnancy were included in estimates for contraceptive services. Receipt was measured for the past year because women using moderately effective methods^§^ need to obtain a prescription from a health care professional each year and women using less effective methods,^¶^ or no method, might benefit from annual counseling to help them assess the suitability of a more effective method and then potentially obtain that method or a prescription. Because women using permanent and long-acting reversible methods** do not necessarily need contraceptive services every year but might benefit from contraceptive counseling, separate estimates were generated for women who were using these methods.

With respect to services for achieving pregnancy, estimates were limited to women with infertility.^††^ Estimates were used to characterize whether these women had ever received advice and whether they had ever received any additional services (infertility testing, ovulation stimulation, surgery to correct blocked tubes, artificial insemination, and other types of medical help).

With respect to STD screening, CDC recommends annual chlamydia testing for all sexually active women aged <25 years, whereas STD testing among men and older women, as well as testing for other STDs, is recommended on the basis of additional risk factors (*1*,*2*,*15*). Accordingly, the prevalence of chlamydia testing was estimated for all women aged <25 years who had oral, anal, or vaginal sex with at least one opposite sex partner in the past year. In contrast, estimates for older women (aged 25–44 years) and all men, as well as estimates for other STDs, were limited to those who were not in a mutually monogamous relationship,^§§^ which was used as a proxy for some of the additional risk factors included in STD testing recommendations (*15*). For human immunodeficiency virus (HIV), because testing is recommended through routine clinical care for all persons aged 13–65 years, unless from a patient population with documented low prevalence of undiagnosed HIV infection (*16*), all adults aged 15–44 years were included in estimates of ever having been tested for HIV outside of blood donation.

For human papillomavirus (HPV), the vaccine was first available in 2006 and recommendations for women include vaccination at age 11–12 years, or at age 13–26 years if not previously vaccinated (*17**,**18*). Accordingly, as in other recent surveillance reports (*32*), overall estimates of having ever received the HPV vaccine were limited to women aged 18–26 years. For cervical cytology, Papanicolaou (Pap) tests are recommended once every 3 years for women aged 21–65 years or, for women aged 30–65 years, once every 5 years if done in combination with HPV testing (*1*,*2*,*19*). Because the HPV questions were not included on the 2013 NHIS questionnaire, estimates were only generated for receipt of a Pap test within the past 3 years. Women aged <21 years were excluded from overall estimates because they fell outside of age-based recommendations, and women aged >44 years were excluded because of the focus of this report on women of reproductive age. Estimates for women aged 18–20 years are provided as a measure of potential overscreening among younger women.

For some preconception care services (counseling before the most recent pregnancy on how to achieve a healthy pregnancy and selected preconception care topics), PRAMS data were used to limit estimates to women with a recent live birth.^¶¶^ For other preconception care services, all women of reproductive age were included in estimates, given the benefit of these services for improving their overall health as well as their pregnancy health. For blood pressure, QFP originally recommended routine screening among persons without recognized hypertension or other risk factors and annual screening for adolescents and prehypertensive adults (i.e., adults with blood pressure 120–139 mm Hg/80–89 mm Hg); for diabetes, QFP originally recommended testing for asymptomatic adults with sustained high blood pressure (either treated or untreated) >135/80 mm Hg (*1*,*20*–*22*). These recommendations have been updated to reduce the frequency of blood pressure screening among persons without risk factors to once every 3–5 years, and to limit diabetes screening to persons aged 40–70 years (*2*,*23*,*24*). Nonetheless, to assess screening according to the recommendations in place at the time, estimates of annual screening were generated; for blood pressure these estimates were among all women aged 18–44 years without a history of hypertension, and for diabetes these estimates were among all women aged 18–44 years with no previous diagnosis and a history of hypertension.

Although QFP follows the USPSTF recommendation to measure weight and height and refer women and men with obesity for intensive counseling and behavioral interventions to promote sustained weight loss (*25*), estimates were made of the proportion of women with obesity who talked with a health care professional about diet as the closest proxy indicator available from the national surveillance data. Similarly, QFP follows the USPSTF recommendation that clinicians ask all adults about their tobacco use, advise them to stop using tobacco, and provide behavioral interventions, along with U.S. Food and Drug Administration–approved pharmacotherapy for cessation if not pregnant (*26*). However, because of availability of national surveillance data, estimates were generated among current smokers for having spoken with a health care professional about their smoking. Because of the lack of a recommended timeframe for obesity and smoking screening and counseling, estimates were generated for receipt in the past year for both of these services.

For influenza and tetanus vaccines, receipt was estimated within the past year and past 10 years, respectively, to match the recommended interval for receipt of these vaccines. All women aged 18–44 years were included in estimates because of the inclusion of all adults in these recommendations (*27*).

Finally, QFP follows the USPSTF recommendation to conduct mammography biennially for women aged 50–74 years and to screen women aged <50 years if other conditions support providing this service for an individual patient (*1*,*2*,*28*). Accordingly, biennial screening estimates were generated for women aged 30–44 years to assess prevalence of screening outside of age-based recommendations. Because receipt of mammography screening is asked only among women aged ≥30 years on the NHIS, women aged 18–29 years are not included in these estimates.

### Data Analysis

All estimates include the weighted prevalence and 95% confidence intervals (CIs) for each indicator, overall and stratified by age, race/ethnicity, family income as a percentage of federal poverty level (FPL), and continuity of insurance coverage. Age categories were specific to each data source (15–19, 20–24, 25–34, and 35–44 years [NSFG]; ≤19, 20–24, 25–34, and ≥35 years [PRAMS]; and 18–19, 20–24, 25–34, and 35–44 years [NHIS]). All three surveys used the same stratifications for race/ethnicity (non-Hispanic white [white], non-Hispanic black [black], Hispanic, and non-Hispanic other or multiple races) and income (≤138% FPL, 139%–250% FPL, 251%–400% FPL, and >400% FPL). Income categories were defined to correspond with the income eligibility maximum for Medicaid insurance coverage in states that expanded Medicaid coverage under the Affordable Care Act (≤138% FPL) and subsidized care provided through clinics receiving grant support through the Title X federal family planning program (≤250% FPL). In assigning respondents to income categories, income for each person was first assigned to the closest whole integer relative to FPL and then placed in the appropriate category. Stratifications for insurance coverage from NSFG and NHIS (continuous insurance coverage in the past year, insurance coverage with gaps in the past year, or no insurance coverage in the past year) differ from the stratifications for insurance coverage from PRAMS (had insurance coverage during the month before conception or did not have insurance coverage during the month before conception).

To provide general guidance on the statistical significance of differences, 95% CIs were compared across strata, with an emphasis on identifying differences between the highest and lowest categories for ordinal variables (i.e., age, income, and insurance coverage). In a report that contains multiple post-hoc comparisons, some statistical differences will occur because of chance. As a result, comparing CIs provides a general idea of the size of the differences versus the standard errors of each estimate and an indication of which differences might be significant. This is typically a conservative approach that might fail to note differences between estimates more often than formal statistical testing (*33*) and was selected to account for precision of estimates while also highlighting large differences (*34*). Lack of comment regarding the difference between any two estimates does not infer no difference exists.

In accordance with the reporting policies of specific surveillance systems, estimates are not reported when outcome data are considered insufficient to produce reliable estimates. For NSFG, estimates are not provided for outcomes with a numerator <5 or a denominator <100. For PRAMS, estimates are not reported for outcomes with a denominator <30; estimates that were based on 30–59 respondents are reported with a footnote stating they might not be reliable and should be used with caution. For NHIS, estimates are not provided for outcomes with a denominator <30 or a relative standard error (RSE)*** ≥50%; estimates with an RSE 30%–49% are reported with a footnote stating they might not be reliable and should be used with caution. All analyses were conducted using population weighted data and statistical software to account for the complex sample design of each survey.

### Presentation of Findings

Results are presented in a manner consistent with the categories of services defined in QFP. For each category of services, selected findings are highlighted in the text and estimates are presented in tables that address the following groups: receipt of contraceptive services, medical services for achieving pregnancy, and STD testing and prevention services; receipt of preconception counseling services; and receipt of preconception care and related preventive health services. For each group of services, estimates of receipt among the population at risk are presented overall and then stratified by age, race/ethnicity, family income, and insurance coverage.

## Results

### Services for Preventing and Achieving Pregnancy

#### Contraceptive Services

Among women aged 15–44 years at risk for unintended pregnancy, less than half (46.5%) received contraceptive services in the past year (Table 1). Among women using a moderately effective contraceptive method (*35*) requiring a prescription from a health care professional, 97.2% received contraceptive services in the past year. However, among women using less effective methods that typically do not require a prescription, and among women not using any contraceptive method, 32.8% and 20.9%, respectively, received contraceptive services in the past year. By comparison, among women using permanent and long-acting reversible methods (i.e., women who do not need contraceptive services annually), 28.5% received contraceptive services in the past year.

**TABLE 1:** Percentage of women and men aged 15–44 years receiving contraceptive, infertility, and STD/HIV testing and prevention services, by age **—** National Survey of Family Growth, 2011–2013 and National Health Interview Survey, 2013

Type of service	Total (15–44 yrs)	15–19 yrs	20–24 yrs	25–34 yrs	35–44 yrs
	% (95% CI)	% (95% CI)	% (95% CI)	% (95% CI)	% (95% CI)
Received contraceptive services in the past yr* (NSFG)					
**Women**					
Among women aged 15–44 yrs at risk for unintended pregnancy^†^	**46.5 (43.8–49.2)**	65.8 (59.5–72.0)	68.2 (63.4–73.0)	52.0 (47.8–56.2)	27.1 (23.3–31.0)
Using sterilization, IUD, or implant	**28.5 (24.8–32.1)**	—^§^	70.5 (59.6–81.4)	39.7 (31.4–48.0)	16.7 (13.1–20.4)
Using a moderately effective method^¶^	**97.2 (95.9–98.6)**	98.4 (96.3–100)	96.6 (93.9–99.3)	96.8 (94.1–99.6)	98.2 (96.4–100)
Using a less effective method**	**32.8 (28.0–37.5)**	39.5 (27.6–51.4)	41.0 (30.6–51.5)	31.6 (25.2–38.0)	23.8 (16.3–31.3)
Using no method	**20.9 (16.1–25.8)**	—	—	26.2 (17.2–35.3)	10.1 (4.8–15.5)
**Men**					
Among men aged 15–44 yrs who had vaginal intercourse in the past yr	**4.5 (3.4–5.6)**	14.5 (9.3–19.8)	4.0 (2.3–5.8)	4.0 (2.5–5.5)	2.8 (1.6–4.0)
**Ever received medical services for achieving pregnancy^††^ (NSFG)**					
**Advice, no additional medical services**					
Among women aged 15–44 yrs	**1.5 (0.9–2.0)**	0.2 (0.0–0.4)	1.9 (0.5–3.3)	2.2 (0.9–3.5)	1.2 (0.6–1.7)
Among women who are infertile^§§^	**3.3 (1.2–5.5)**	—	—	—	—
**Medical services beyond advice**					
Among women aged 15–44 yrs	**5.9 (4.8–7.0)**	—	1.1 (0.3–2.0)	6.3 (4.5–8.0)	10.8 (8.4–13.1)
Among women who are infertile	**27.7 (19.4–36.1)**	—	—	—	—
**Received STD screening services in the past yr (NSFG)**					
**Women**					
**Chlamydia screening**					
Among women aged 15–24 yrs who had oral, anal, or vaginal sex with a male partner in the past yr	**37.5 (32.8–42.3)^¶¶^**	30.6 (24.2–37.1)	41.2 (35.5–46.9)	N/A	N/A
Among women aged 15–44 yrs who had oral, anal, or vaginal sex with a male partner in the past yr and were not in a mutually monogamous relationship***	**45.3 (39.8–50.7)**	35.6 (25.5–45.6)	49.9 (41.1–58.7)	52.9 (42.6–63.2)	36.2 (29.6–42.8)
Among women aged 15–44 yrs who were pregnant in the past yr	**48.6 (41.6–55.7)**	—	61.7 (51.1–72.4)	46.1 (36.2–56.1)	—
**Screening for any STD other than chlamydia**					
Among women aged 15–44 yrs who had oral, anal, or vaginal sex with a male partner in the past yr and were not in a mutually monogamous relationship***	**48.8 (43.2–54.5)**	36.8 (26.5–47.1)	54.7 (45.9–63.5)	58.3 (48.7–67.9)	37.4 (30.4–44.4)
**Men**					
**Screening for any STD**					
Among men aged 15–44 yrs who had oral, anal, or vaginal sex with a female partner in the past yr and were not in a mutually monogamous relationship***	**32.5 (29.0–36.0)**	23.6 (18.0–29.2)	34.5 (28.3–40.7)	41.0 (34.5–47.6)	26.3 (19.0–33.6)
**STD services ever received (NSFG and NHIS)**					
**Women**					
**Ever tested for HIV**					
Among women aged 15–44 yrs (NSFG)^†††^	**62.2 (59.4–65.0)**	19.6 (15.1–24.0)	53.2 (46.3–60.0)	76.2 (73.0–79.3)	72.6 (68.7–76.4)
**Ever received the hepatitis B vaccine**					
Among women aged 18–44 yrs (NHIS)	**46.8 (45.2–48.3)^§§§^**	53.4 (46.2–60.5)^¶¶¶^	55.8 (52.0–59.6)	50.4 (48.0–52.7)	36.8 (34.6–39.1)
**Ever received at least three doses of the hepatitis B vaccine**					
Among women aged 18–44 yrs (NHIS)	**39.8 (38.2–41.3)^§§§^**	46.9 (39.7–54.2)^¶¶¶^	47.2 (43.2–51.1)	43.0 (40.6–45.4)	31.2 (29.0–33.5)
**Ever received the HPV vaccine**					
Among women aged 18–26 yrs (NHIS)	36.8 (33.9–39.7)****	43.6 (36.7–50.6)^¶¶¶^	37.4 (33.9– 41.1)	27.9 (23.0–33.4)^††††^	N/A
**Ever received at least three doses of the HPV vaccine**					
Among women aged 18–26 yrs (NHIS)	24.8 (22.4–27.5)****	31.6 (25.3–38.7)^¶¶¶^	24.4 (21.3–27.7)	19.2 (14.8–24.4)^††††^	N/A
**Men**					
**Ever tested for HIV**					
Among men aged 15–44 yrs (NSFG)^†††^	**46.8 (43.7–49.9)**	15.4 (12.4–18.3)	41.7 (36.3–47.2)	54.9 (50.8–59.0)	57.4 (52.5–62.3)

**Abbreviations**: CI = confidence interval; HIV = human immunodeficiency virus; HPV = human papilloma virus; IUD = intrauterine device; N/A = not applicable; NHIS = National Health Interview Survey; NSFG = National Survey on Family Growth; STD = sexually transmitted disease.

* For women, contraceptive services include receiving a birth control method or a prescription, receiving a checkup for birth control, receiving counseling about birth control, receiving a sterilizing operation, receiving counseling about a sterilizing operation, receiving emergency contraception, and receiving counseling about emergency contraception. For men, contraceptive services include receiving information or advice about using condoms, information or advice about female methods of birth control, and information or advice about getting a vasectomy.

^†^ Women were considered at risk for unintended pregnancy if they had ever had vaginal intercourse; were neither pregnant, seeking pregnancy, nor postpartum; and neither they nor their partner were noncontraceptively sterile. Postpartum women were identified to have completed a recent pregnancy ≤2.5 months before interview according to the coding specifications (https://www.cdc.gov/nchs/data/nsfg/nsfg_2011-2013_app3a_femresprecodespecs_v2.pdf).

^§^ Does not meet reliability standards.

^¶^ Includes injectable, patch, ring, pill, and diaphragm.

** Includes condoms, withdrawal, morning-after pill, foam, sponge, suppository or insert, jelly or cream, periodic abstinence, and other (unspecified) methods.

^††^ Medical services to help women achieve pregnancy include infertility testing (for herself or her male partner), ovulation stimulation, surgery to correct blocked tubes, artificial insemination, and other types of medical help.

^§§^ Infertility is defined as a lack of pregnancy in the 12 months before survey, despite having had unprotected sexual intercourse in each of those months with the same husband or cohabiting partner.

^¶¶^ Includes women aged 15–24 years only.

*** Had more than one opposite sex partner in the past year, or had an opposite sex partner with other partners in the past year.

^†††^ Includes testing outside of a blood donation only.

^§§§^ Includes women aged 18–44 years only.

^¶¶¶^ Includes women aged 18–19 years only.

**** Includes women aged 18–26 years only because the HPV vaccine is not recommended for women aged >26 years.

^††††^ Includes women aged 25–26 years only.

Among women using all categories of methods combined, across age categories receipt of contraceptive services was higher among younger than among older women (range: 65.8% and 68.2% for women aged 15–19 and 20–24 years, respectively, to 50.2% and 27.1% for women aged 25–34 years and 35–44 years, respectively) (Table 1). By race/ethnicity (Table 2) and family income (Table 3), CIs overlapped across all categories, although by income the lowest estimates occurred among women with family income ≤138% FPL (43.7%) and 139%–250% FPL (43.4%) and the highest estimates occurred among women with family income 251%–400% FPL (48.0%) and >400% FPL (53.0%). By insurance coverage, receipt of contraceptive services in the past year was higher among women who had continuous insurance coverage (49.7%) or coverage with gaps (50.9%) compared with those with no insurance (29.1%) (Table 4).

**TABLE 2:** Percentage of women and men aged 15–44 years receiving contraceptive, infertility, and STD/HIV testing and prevention services, by race/ethnicity **—** National Survey of Family Growth, 2011–2013 and National Health Interview Survey, 2013

Type of service	Non-Hispanic white	Non-Hispanic black	Hispanic	Non-Hispanic other or multiple races
	% (95% CI)	% (95% CI)	% (95% CI)	% (95% CI)
Received contraceptive services in past yr* (NSFG)				
**Women**				
Among women aged 15–44 yrs at risk for unintended pregnancy^†^	47.9 (44.3–51.6)	44.0 (38.8–49.1)	43.8 (38.6–49.0)	47.0 (40.0–54.0)
Among women using sterilization, IUD, or implant	28.7 (24.2–33.3)	25.8 (17.7–34.0)	28.1 (21.4–34.7)	—^§^
Among women using a moderately effective method^¶^	98.1 (96.7–99.5)	94.4 (87.4–100)	96.3 (93.3–99.3)	—
Among women using a less effective method**	32.2 (24.7–39.7)	34.8 (27.1–42.4)	39.2 (31.3–47.1)	—
Among women using no method	19.6 (10.9–28.3)	27.9 (18.5–37.3)	23.2 (13.0–33.5)	—
**Men**				
Among men aged 15–44 yrs who had vaginal intercourse in the past yr	4.3 (2.6–6.0)	5.3 (3.4–7.2)	4.6 (3.0–6.2)	4.7 (1.7–7.8)
**Ever received medical services for achieving pregnancy^††^ (NSFG)**				
**Advice, no additional medical services**				
Among women aged 15–44 yrs	1.7 (1.0–2.5)	0.9 (0.4–1.5)	1.4 (0.4–2.5)	0.8 (0.0–1.6)
**Medical services beyond advice**				
Among women aged 15–44 yrs	7.4 (5.7–9.1)	3.1 (1.3–4.8)	2.8 (1.7–3.8)	7.4 (2.7–12.2)
**Received STD screening services in the past yr (NSFG)**				
**Women**				
**Chlamydia screening**				
Among women aged 15–24 yrs who had oral, anal, or vaginal sex with male partner in the past yr	31.6 (25.3–37.9)	60.5 (50.2–70.8)	36.7 (29.3–44.1)	—
Among women aged 15–44 yrs who had oral, anal, or vaginal sex with a male partner in the past yr and were not in a mutually monogamous relationship^§§^	42.2 (34.2–50.3)	51.6 (42.0–61.2)	52.1 (44.2–60.0)	—
Among women aged 15–44 yrs who were pregnant in the past yr	38.5 (30.1–46.9)	83.1 (75.3–90.9)	44.0 (32.2–55.8)	—
**Screening for any STD other than chlamydia**				
Among women aged 15–44 yrs who had oral, anal, or vaginal sex with a male partner in the past yr and were not in a mutually monogamous relationship^§§^	44.0 (35.6–52.5)	61.1 (53.7–68.4)	55.5 (46.7–64.3)	—
**Men**				
**Screening for any STD**				
Among men aged 15–44 yrs who had oral, anal, or vaginal sex with a female partner in the past yr were not in a mutually monogamous relationship^§§^	26.5 (20.9–32.2)	44.8 (37.6–51.9)	37.0 (28.2–45.7)	—
**STD services ever received (NSFG and NHIS)**				
**Women**				
**Ever tested for HIV**				
Among women aged 15–44 yrs (NSFG)^¶¶^	57.5 (53.8–61.2)	80.5 (76.4–84.5)	66.0 (62.1–69.9)	55.2 (49.8–60.6)
**Ever received the hepatitis B vaccine**				
Among women aged 18–44 yrs (NHIS)	49.7 (47.6–51.9)	43.4 (40.0–46.9)	39.1 (35.9–42.5)	49.4 (44.8–54.0)
**Ever received at least three doses of the hepatitis B vaccine**				
Among women aged 18–44 yrs (NHIS)	43.4 (41.3–45.6)	36.6 (33.2–40.2)	29.5 (26.4–32.7)	42.9 (38.3–47.7)
**Ever received the HPV vaccine**				
Among women aged 18–26 yrs (NHIS)***	41.2 (37.2–45.3)	30.9 (25.4–36.9)	30.8 (25.5–36.8)	29.0 (20.3–39.4)
**Ever received at least three doses of the HPV vaccine**				
Among women aged 18–26 yrs (NHIS)***	30.5 (26.9–34.4)	14.5 (10.4–19.7)	17.4 (13.2–22.5)	20.0 (13.7–28.3)
**Men**				
**Ever tested for HIV**				
Among men aged 15–44 yrs (NSFG)^¶¶^	42.9 (39.2–46.6)	68.6 (64.1–73.1)	44.2 (38.6–49.8)	49.0 (41.6–56.4)

**Abbreviations:** CI = confidence interval; HIV = human immunodeficiency virus; HPV = human papilloma virus; IUD = intrauterine device; NHIS = National Health Interview Survey; NSFG = National Survey of Family Growth; STD = sexually transmitted disease.

* For women, contraceptive services include receiving a birth control method or a prescription, receiving a checkup for birth control, receiving counseling about birth control, receiving a sterilizing operation, receiving counseling about a sterilizing operation, receiving emergency contraception, and receiving counseling about emergency contraception. For men, contraceptive services include receiving information or advice about using condoms, information or advice about female methods of birth control, and information or advice about getting a vasectomy.

^†^ Women were considered at risk for unintended pregnancy if they had ever had vaginal intercourse; were neither pregnant, seeking pregnancy, nor postpartum; and neither they nor their partner were noncontraceptively sterile. Postpartum women were identified to have completed a recent pregnancy ≤2.5 months before interview according to the coding specifications (https://www.cdc.gov/nchs/data/nsfg/nsfg_2011-2013_app3a_femresprecodespecs_v2.pdf).

^§^ Does not meet reliability standards.

^¶^ Includes injectable, patch, ring, pill, and diaphragm.

** Includes condoms, withdrawal, morning-after pill, foam, sponge, suppository or insert, jelly or cream, periodic abstinence, and other (unspecified) methods.

^††^ Medical services to help women achieve pregnancy include infertility testing (for herself or her male partner), ovulation stimulation, surgery to correct blocked tubes, artificial insemination, and other types of medical help.

^§§^ Had more than one opposite sex partner in the past year, or had an opposite sex partner with other partners in the past year.

^¶¶^ Includes testing outside of a blood donation only.

*** Includes women aged 18–26 years only because the HPV vaccine is not recommended for women aged >26 years.

**TABLE 3:** Percentage of women and men aged 15–44 years receiving contraceptive, infertility, and STD/HIV testing and prevention services, by family income **—** National Survey of Family Growth, 2011–2013 and National Health Interview Survey, 2013

Type of service	≤138% FPL	139%–250% FPL	251%–400% FPL	>400% FPL
	% (95% CI)	% (95% CI)	% (95% CI)	% (95% CI)
Received contraceptive services in past yr* (NSFG)				
**Women**				
Among women aged 15–44 yrs at risk for unintended pregnancy^†^	43.7 (40.4–47.1)	43.4 (37.8–49.1)	48.0 (43.5–52.4)	53.0 (46.6–59.4)
Among women using sterilization, IUD, or implant	29.7 (24.0–35.3)	28.7 (20.7–36.7)	26.9 (18.2–35.7)	27.3 (19.4–35.1)
Among women using a moderately effective method^§^	95.0 (91.6–98.3)	98.7 (97.3–100)	96.5 (92.3–100)	99.2 (98.3–100)
Among women using a less effective method^¶^	34.7 (27.4–41.9)	27.9 (18.6–37.2)	31.1 (22.4–39.8)	36.0 (23.5–48.6)
Among women using no method	20.3 (13.5–27.2)	18.9 (10.3–27.5)	—**	—
**Men**				
Among men aged 15–44 yrs who had vaginal intercourse in the past yr	6.3 (4.5–8.0)	3.6 (2.1–5.2)	3.9 (2.3–5.5)	4.1 (2.2–6.0)
**Ever received medical services for achieving pregnancy^††^ (NSFG)**				
**Advice, no additional medical services**				
Among women aged 15–44 yrs	1.1 (0.4–1.7)	0.7 (0.3–1.2)	3.1 (1.4–4.8)	1.5 (0.8–2.3)
**Medical services beyond advice**				
Among women aged 15–44 yrs	2.1 (1.3–2.9)	5.0 (3.4–6.7)	7.1 (4.7–9.6)	12.4 (8.5–16.4)
**Received STD screening services in the past yr (NSFG)**				
**Women**				
**Chlamydia screening**				
Among women aged 15–24 yrs who had oral, anal, or vaginal sex with a male partner in the past yr	39.6 (34.1–45.1)	38.2 (29.6–46.7)	32.9 (22.6–43.3)	35.6 (21.1–50.2)
Among women aged 15–44 yrs who had oral, anal, or vaginal sex with a male partner in the past yr and were not in a mutually monogamous relationship^§§^	44.1 (37.0–51.2)	49.0 (40.1–57.8)	47.3 (34.3–60.3)	41.6 (21.1–62.0)
Among women aged 15–44 yrs who were pregnant in the past yr	62.4 (54.8–70.0)	40.5 (27.5–53.4)	—	—
**Screening for any STD other than chlamydia**				
Among women aged 15–44 yrs who had oral, anal, or vaginal sex with a male partner in the past yr and were not in a mutually monogamous relationship^§§^	50.2 (42.9–57.4)	52.3 (43.2–61.3)	45.0 (30.9–59.0)	42.8 (22.9–62.8)
**Men**				
**Screening for any STD**				
Among men aged 15–44 yrs who had oral, anal, or vaginal sex with a female partner in the past yr and were not in a mutually monogamous relationship^§§^	39.9 (33.9–45.8)	36.5 (30.2–42.9)	25.1 (16.9–33.2)	25.2 (18.3–32.1)
**STD services ever received (NSFG and NHIS)**				
**Women**				
**Ever tested for HIV**				
Among women aged 15–44 yrs (NSFG)^¶¶^	65.1 (61.4–68.7)	62.7 (58.3–67.1)	57.0 (50.9–63.1)	61.3 (55.8–66.7)
**Ever received the hepatitis B vaccine**				
Among women aged 18–44 yrs (NHIS)	43.4 (40.7–46.1)	45.8 (42.3–49.3)	47.5 (44.2–50.8)	50.3 (47.4–53.2)
**Ever received at least three doses of the hepatitis B vaccine**				
Among women aged 18–44 yrs (NHIS)	35.1 (32.5–37.8)	39.2 (35.7–42.8)	40.3 (36.8–43.8)	44.2 (41.2–47.2)
**Ever received the HPV vaccine**				
Among women aged 18–26 yrs (NHIS)***	34.8 (30.8–39.0)	29.6 (24.2–35.8)	36.4 (29.8–43.5)	49.3 (41.7–56.9)
**Ever received at least three doses of the HPV vaccine**				
Among women aged 18–26 yrs (NHIS)***	22.3 (18.7–26.3)	17.3 (13.1–22.5)	26.1 (20.5–32.5)	37.1 (29.8–45.1)
**Men**				
**Ever tested for HIV**				
Among men aged 15–44 yrs (NSFG)^¶¶^	46.1 (41.5–50.8)	45.2 (40.0–50.5)	46.9 (41.5–52.2)	49.0 (44.0–54.0)

**Abbreviations:** CI = confidence interval; FPL = federal poverty level; HIV = human immunodeficiency virus; HPV = human papilloma virus; IUD = intrauterine device; NHIS = National Health Interview Survey; NSFG = National Survey of Family Growth; STD = sexually transmitted disease.

* For women, contraceptive services include receiving a birth control method or a prescription, receiving a checkup for birth control, receiving counseling about birth control, receiving a sterilizing operation, receiving counseling about a sterilizing operation, receiving emergency contraception, and receiving counseling about emergency contraception. For men, contraceptive services include receiving information or advice about using condoms, information or advice about female methods of birth control, and information or advice about getting a vasectomy.

^†^ Women were considered at risk for unintended pregnancy if they had ever had vaginal intercourse; were neither pregnant, seeking pregnancy, nor postpartum; and neither they nor their partner were noncontraceptively sterile. Postpartum women were identified to have completed a recent pregnancy ≤2.5 months before interview according to the coding specifications (https://www.cdc.gov/nchs/data/nsfg/nsfg_2011-2013_app3a_femresprecodespecs_v2.pdf).

^§^ Includes injectable, patch, ring, pill, and diaphragm.

^¶^ Includes condoms, withdrawal, morning-after pill, foam, sponge, suppository or insert, jelly or cream, periodic abstinence, and other (unspecified) methods.

** Does not meet reliability standards.

^††^ Medical services to help women achieve pregnancy include infertility testing (for herself or her male partner), ovulation stimulation, surgery to correct blocked tubes, artificial insemination, and other types of medical help.

^§§^ Had more than one opposite sex partner in the past year, or had an opposite sex partner with other partners in the past year.

^¶¶^ Includes testing outside of a blood donation only.

*** Includes women aged 18–26 years only because the HPV vaccine is not recommended for women aged >26 years.

**TABLE 4:** Percentage of women and men aged 15–44 years receiving contraceptive, infertility, and STD/HIV testing and prevention services, by continuity of health insurance coverage during the past year **—** National Survey of Family Growth, 2011–2013 and National Health Interview Survey, 2013

Type of service	Had insurance coverage continuously during the past yr	Had insurance coverage with gaps during the past yr	Did not have any insurance coverage during the past yr
	% (95% CI)	% (95% CI)	% (95% CI)
Received contraceptive services in past yr* (NSFG)			
**Women**			
Among women aged 15–44 yrs at risk for unintended pregnancy^†^	49.7 (46.2–53.2)	50.9 (45.6–56.3)	29.1 (23.3–34.8)
Among women using sterilization, IUD, or implant	30.2 (26.1–34.3)	38.0 (26.4–49.5)	13.5 (6.7–20.2)
Among women using a moderately effective method^§^	99.0 (98.4–99.6)	92.5 (85.0–100)	88.7 (79.8–97.6)
Among women using a less effective method^¶^	33.0 (26.5–39.5)	42.6 (29.5–55.7)	25.3 (16.7–33.9)
Among women using no method	22.6 (15.7–29.4)	—**	8.2 (0.20–16.2)
**Men**			
Among men aged 15–44 yrs who had vaginal intercourse in the past yr	5.2 (3.7–6.6)	4.1 (1.9–6.4)	2.7 (1.5–3.8)
**Ever received medical services for achieving pregnancy^††^ (NSFG)**			
**Advice, no additional medical services**			
Among women aged 15–44 yrs	1.5 (0.8–2.1)	2.3 (0.9–3.8)	1.0 (0.2–1.7)
**Medical services beyond advice**			
Among women aged 15–44 yrs	7.3 (5.8–8.8)	2.1 (1.0–3.2)	2.0 (1.0–2.9)
**Received STD screening services in the past yr (NSFG)**			
**Women**			
**Chlamydia screening**			
Among women aged 15–24 yrs who had oral, anal or vaginal sex with a male partner in the past yr	39.0 (33.6–44.3)	36.7 (27.6–45.8)	31.4 (20.9–41.9)
Among women aged 15–44 yrs who had oral, anal, or vaginal sex with a male partner in the past yr and were not in a mutually monogamous relationship^§§^	46.4 (39.8–53.1)	46.2 (34.8–57.5)	40.6 (30.0–51.3)
Among women aged 15–44 yrs who were pregnant in the past yr	49.1 (40.3–57.9)	—	—
**Screening for any STD other than chlamydia**			
Among women aged 15–44 yrs who had oral, anal, or vaginal sex with a male partner in the past yr and were not in a mutually monogamous relationship^§§^	49.4 (42.8–55.9)	50.8 (39.3–62.2)	45.6 (34.6–56.6)
**Men**			
**Screening for any STD**			
Among men aged 15–44 yrs who had oral, anal, or vaginal sex with a female partner in the past yr and were not in a mutually monogamous relationship^§§^	32.8 (28.3–37.2)	35.4 (24.7–46.2)	29.9 (22.6–37.3)
**STD services ever received (NSFG and NHIS)**			
**Women**			
**Ever tested for HIV**			
Among women aged 15–44 yrs (NSFG)^¶¶^	60.3 (57.1–63.6)	73.2 (68.3–78.1)	64.6 (59.8–69.4)
**Ever received the hepatitis B vaccine**			
Among women aged 18–44 yrs (NHIS)	48.6 (46.8–50.5)	50.7 (46.3–55.1)	35.6 (31.9–39.4)
**Ever received at least three doses of the hepatitis B vaccine**			
Among women aged 18–44 yrs (NHIS)	41.7 (39.9–43.5)	43.1 (38.7–47.8)	28.1 (24.5–32.0)
**Ever received the HPV vaccine**			
Among women aged 18–26 yrs (NHIS)***	40.8 (37.4–44.4)	33.5 (26.7–41.1)	21.5 (16.2–27.9)
**Ever received at least three doses of the HPV vaccine**			
Among women aged 18–26 yrs (NHIS)***	28.8 (25.6–32.2)	18.1 (13.1–24.4)	12.8 (8.4–18.9)
**Men**			
**Ever tested for HIV**			
Among men aged 15–44 yrs (NSFG)^¶¶^	45.9 (42.7–49.1)	51.3 (45.4–57.3)	49.1 (43.8–54.3)

**Abbreviations:** CI = confidence interval; HIV = human immunodeficiency virus; HPV = human papilloma virus; IUD = intrauterine device; NHIS = National Health Interview Survey; NSFG = National Survey of Family Growth; STD = sexually transmitted disease.

* For women, contraceptive services include receiving a birth control method or a prescription, receiving a checkup for birth control, receiving counseling about birth control, receiving a sterilizing operation, receiving counseling about a sterilizing operation, receiving emergency contraception, and receiving counseling about emergency contraception. For men, contraceptive services include receiving information or advice about using condoms, information or advice about female methods of birth control, and information or advice about getting a vasectomy.

^†^ Women were considered at risk for unintended pregnancy if they had ever had vaginal intercourse; were neither pregnant, seeking pregnancy, nor postpartum; and neither they nor their partner were noncontraceptively sterile. Postpartum women were identified to have completed a recent pregnancy ≤2.5 months before interview according to the coding specifications (https://www.cdc.gov/nchs/data/nsfg/nsfg_2011-2013_app3a_femresprecodespecs_v2.pdf).

^§^ Includes injectable, patch, ring, pill, and diaphragm.

^¶^ Includes condoms, withdrawal, morning-after pill, foam, sponge, suppository or insert, jelly or cream, periodic abstinence, and other (unspecified) methods.

** Does not meet reliability standards.

^††^ Medical services to help women achieve pregnancy include infertility testing, ovulation stimulation, surgery to correct blocked tubes, artificial insemination, and other types of medical help.

^§§^ Had more than one opposite sex partner in the past year, or had an opposite sex partner with other partners in the past year.

^¶¶^ Includes testing outside of a blood donation only.

*** Includes women aged 18–26 years only because the HPV vaccine is not recommended for women aged >26 years.

Among men aged 15–44 years who had vaginal intercourse in the past year, 4.5% received contraceptive services, including counseling and information about male and female methods of birth control in the past year. By age, receipt of contraceptive services was higher among men aged 15–19 years (14.5%) compared with men aged 20–44 years (range: 2.8%–4.0%) (Table 1).

#### Infertility: Medical Services Related to Achieving Pregnancy

Among women aged 15–44 years, irrespective of infertility status, 1.5% had ever received advice for achieving pregnancy from a medical professional, with no additional services for achieving pregnancy;^†††^ 5.9% had ever received services beyond advice for achieving pregnancy. Ever use of medical services beyond advice increased with age and was highest among women aged 35–44 years (10.8%) (Table 1). By race/ethnicity, ever use of medical services beyond advice was higher among white (7.4%) than among black (3.1%) and Hispanic (2.8%) women (Table 2). By income, use of medical services beyond advice was higher among women with family income >400% FPL (12.4%) compared with ≤138% FPL (2.1%) or 139%–250% FPL (5.0%) (Table 3). By insurance coverage, ever use of medical services beyond advice was higher among women who had continuous insurance coverage during the past year (7.3%) compared with those who had gaps in coverage (2.1%) or no coverage (2.0%) (Table 4). Among women with infertility, 3.3% had ever received advice for achieving pregnancy with no additional medical services and 27.7% had received medical services beyond advice for achieving pregnancy (Table 1).

### STD Screening and Prevention Services

#### Screening for STDs and HIV

Despite recommendations for annual chlamydia screening in each of the following groups, testing in the past year was only reported by 37.5% of all women aged 15–24 years who had oral, anal, or vaginal sex with a male partner in the past year, 45.3% of women aged 15–44 years who were not in a mutually monogamous relationship, and 48.6% of women aged 15–44 years who were pregnant in the past year (Table 1). Testing in the past year for STDs other than chlamydia was reported by 48.8% of women aged 15–44 years who were not in a mutually monogamous relationship. Similarly, despite recommendations for HIV testing through routine clinical care among all adults, only 62.2% of women aged 15–44 years had ever been tested outside of blood donation (Table 1). By race/ethnicity, for each of the above measures, except for chlamydia testing among women who were not in a mutually monogamous relationship, the prevalence of testing was highest among black women followed by Hispanic women and white women (Table 2).

For men aged 15–44 years, 32.5% of those not in a mutually monogamous relationship had been tested for any STD in the past year; 46.8% of all men had ever been tested for HIV outside of blood donation (Table 1). For both of these outcomes, the prevalence of testing differed by race/ethnicity; black men had the highest prevalence followed by Hispanic men and white men (Table 2).

#### Receipt of Vaccines to Prevent STDs

For the hepatitis B vaccine, 46.8% and 39.8% of all women aged 18–44 years had ever received any or at least three doses, respectively. For the HPV vaccine, 36.8% and 24.8% of all women aged 18–26 years had ever received any or at least three doses, respectively (Table 1). For both vaccines, receipt of any or at least three doses decreased across age groups and was higher among white women compared with black and Hispanic women (Table 2). By income, receipt of any or at least three doses of both vaccines was higher among women with family income >400% FPL compared with ≤138% FPL (Table 3). By insurance coverage, for both vaccines the percentage of women receiving any or at least three doses was higher among those who had continuous insurance in the past year compared with those who had no coverage (Table 4).

### Preconception Care and Related Preventive Health Services

#### Counseling Services Among Women With a Recent Live Birth

Among women with a recent live birth in selected states, 33.2% talked with a health care professional about how to improve their health before their most recent pregnancy (Table 5). This percentage increased with age (25.9% and 25.2%, respectively, for women aged ≤19 years and 20–24 years and 35.9% and 37.8%, respectively, for women aged 25–34 and ≥35 years). By race/ethnicity, this percentage was higher among white (35.2%) compared with black (30.0%) and Hispanic (26.0%) women (Table 6). By income, this percentage was higher among women with family income >400% FPL (46.2%) compared with 251%–400% FPL (36.1%), 139%–250% FPL (26.8%), and ≤138% FPL (23.8%) (Table 7). By insurance coverage, this percentage was higher among women who had health insurance during the month before pregnancy (37.6%) compared with those who did not (14.5%) (Table 8).

**TABLE 5:** Percentage of women with a recent live birth receiving health care services before pregnancy, by age **—** Pregnancy Risk Assessment Monitoring System, selected states, 2011–2012

Type of service	Total	≤19 yrs		20–24 yrs		25–34 yrs		≥35 yrs	
	% (95% CI)	% (95% CI)		% (95% CI)		% (95% CI)		% (95% CI)	
Reported talking with a doctor, nurse, or other health care worker about how to improve her health before pregnancy*									
Among women with a recent live birth	**33.2 (32.0–34.4)**	25.9 (21.7–30.6)		25.2 (22.9–27.7)		35.9 (34.3–37.6)		37.8 (34.9–40.8)	
**Reported talking with a doctor, nurse, or other health care worker before pregnancy**									
**About achieving a healthy weight before pregnancy^†^**									
Among women with a recent live birth	**62.9 (60.3–65.5)**		73.0 (61.6–82.0)		64.8 (57.5–71.4)		61.8 (58.3–65.1)		62.2 (56.5–67.7)
Among underweight/normal weight women (BMI ≤24.9)	**58.3 (54.7–61.8)**		71.7 (57.5–82.6)^§^		60.7 (50.3–70.2)		56.2 (51.5–60.8)		58.1 (50.3–65.5)
Among overweight women (BMI 25.0–29.9)	**65.7 (60.3–70.8)**		—^¶^		66.9 (52.4–78.7)^§^		66.7 (59.7–73.1)		62.4 (50.8–72.6)
Among obese women (BMI >30.0)	**72.4 (66.7–77.4)**		—		71.1 (55.4–83.0)		70.9 (63.4–77.4)		75.0 (62.3–84.4)
**About taking vitamins with folic acid before pregnancy^†^**									
Among women with a recent live birth	**81.2 (79.1–83.2)**		53.9 (42.4–65.0)		60.7 (53.6–67.3)		86.3 (83.9–88.5)		89.1 (85.1–92.1)
About the effects of smoking during pregnancy on a baby**									
Among women with a recent live birth	**58.2 (55.6–60.8)**		85.4 (75.6–91.7)		71.7 (64.7–77.8)		55.1 (51.6–58.5)		49.1 (43.4–54.9)
Among women who reported any level of smoking in the 3 mos before pregnancy	**71.5 (65.3–77.0)**		—		78.7 (65.5–87.8)		69.3 (60.7–76.7)		60.6 (42.5–76.1)^§^
Among women who reported not smoking in the 3 mos before pregnancy	**55.1 (52.1–58.0)**		88.0 (76.4–94.4)		68.5 (60.0–76.0)		52.0 (48.2–55.8)		47.8 (41.8–53.9)
About the effects of drinking alcohol during pregnancy on a baby**									
Among women with a recent live birth	**60.3 (57.7–62.9)**		86.8 (77.2–92.8)		72.9 (66.0–78.9)		57.1 (53.6–60.5)		52.5 (46.8–58.2)
Among women who reported drinking one or more alcoholic drinks per week on average in the 3 mos before pregnancy	**54.2 (49.2–59.1)**		—		76.4 (60.6–87.2)^§^		52.0 (45.9–58.0)		48.5 (37.6–59.5)
Among women who reported drinking less than one alcoholic drink per week on average in the 3 mos before pregnancy	**62.8 (59.7–65.9)**		87.3 (77.0–93.3)		72.0 (64.1–78.7)		59.7 (55.5–63.8)		54.0 (47.3–60.7)

**Abbreviations:** BMI = body mass index calculated as weight (kg)/(height [m])^2^, using self-reported height and weight; CI = confidence interval.

* Data from eight states (Hawaii, Maine, Maryland, Michigan, Minnesota, New Jersey, Utah, and West Virginia).

^†^ Data from six states (Maryland, Michigan, Nebraska, New Jersey, Tennessee, and Vermont).

^§^ Estimate might not be reliable and should be used with caution; represents 30–59 respondents.

^¶^ Does not meet reliability standards.

** Data from five states (Maryland, Michigan, Nebraska, New Jersey, and Tennessee).

**TABLE 6:** Percentage of women with a recent live birth receiving health care services before pregnancy, by race/ethnicity **—** Pregnancy Risk Assessment Monitoring System, selected states, 2011–2012

Type of service	Non-Hispanic white	Non-Hispanic black	Hispanic	Non-Hispanic other or multiple races
	% (95% CI)	% (95% CI)	% (95% CI)	% (95% CI)
Reported talking with a doctor, nurse, or other health care worker about how to improve her health before pregnancy*				
Among women with a recent live birth	35.2 (33.6–36.8)	30.0 (27.1–33.2)	26.0 (22.9–29.3)	34.9 (31.8–38.0)
**Reported talking with a doctor, nurse, or other health care worker before pregnancy**				
**About achieving a healthy weight before pregnancy^†^**				
Among women with a recent live birth	58.4 (54.8–62.0)	66.3 (60.3–71.8)	76.6 (69.2–82.6)	70.0 (62.6–76.5)
Among underweight/normal weight women (BMI ≤24.9)	53.4 (48.7–58.1)	60.9 (51.1–69.9)	77.8 (67.3–85.7)	66.7 (57.5–74.8)
Among overweight women (BMI 25.0–29.9)	63.1 (55.6–69.9)	65.2 (53.1–75.5)	70.9 (56.2–82.2)^§^	76.2 (59.4–87.5)^§^
Among obese women (BMI >30.0)	69.2 (60.7–76.6)	73.6 (63.8–81.6)	82.2 (64.2–92.3)^§^	77.6 (53.7–91.2)^§^
**About taking vitamins with folic acid before pregnancy^†^**				
Among women with a recent live birth	88.2 (85.6–90.4)	60.5 (54.7–66.0)	72.8 (65.8–78.9)	87.4 (81.3–91.7)
**About the effects of smoking during pregnancy on a baby** ^¶^				
Among women with a recent live birth	50.3 (46.7–53.9)	74.6 (69.0–79.4)	75.1 (67.9–81.1)	54.1 (46.3–61.6)
Among women who reported any level of smoking in the 3 mos before pregnancy	67.0 (59.1–74.1)	81.0 (67.1–89.9)	—**	—
Among women who reported not smoking in the 3 mos before pregnancy	45.7 (41.7–49.8)	72.6 (66.3–78.1)	73.9 (66.2–80.3)	52.7 (44.7–60.5)
**About the effects of drinking alcohol during pregnancy on a baby** ^¶^				
Among women with a recent live birth	52.4 (48.8–56.0)	75.5 (70.0–80.3)	78.0 (71.1–83.7)	57.1 (49.3–64.5)
Among women who reported drinking one or more alcoholic drinks per week on average in the 3 mos before pregnancy	48.2 (42.5–54.0)	81.4 (68.2–89.9)	77.7 (57.7–89.9)^§^	—
Among women who reported drinking less than one alcoholic drink per week on average in the 3 mos before pregnancy	54.9 (50.3–59.5)	74.3 (68.1–79.7)	78.1 (70.6–84.1)	57.5 (49.2–65.4)

**Abbreviations:** BMI = body mass index calculated as weight (kg)/(height [m])^2^, using self-reported height and weight; CI = confidence interval.

* Data from eight states (Hawaii, Maine, Maryland, Michigan, Minnesota, New Jersey, Utah, and West Virginia).

^†^ Data from six states (Maryland, Michigan, Nebraska, New Jersey, Tennessee, and Vermont).

^§^ Estimate might not be reliable and should be used with caution; represents 30–59 respondents.

^¶^ Data from five states (Maryland, Michigan, Nebraska, New Jersey, and Tennessee).

** Does not meet reliability standards.

**TABLE 7:** Percentage of women with a recent live birth receiving health care services before pregnancy, by family income **—** Pregnancy Risk Assessment Monitoring System, selected states 2011–2012

Type of service	≤138% FPL	139%–250% FPL	251%–400% FPL	>400% FPL
	% (95% CI)	% (95% CI)	% (95% CI)	% (95% CI)
Reported talking with a doctor, nurse, or other health care worker about how to improve her health before pregnancy*				
Among women with a recent live birth	23.8 (22.1–25.7)	26.8 (24.1–29.7)	36.1 (32.2–40.2)	46.2 (44.0–48.5)
**Reported talking with a doctor, nurse, or other health care worker before pregnancy**				
**About achieving a healthy weight before pregnancy^†^**				
Among women with a recent live birth	68.8 (62.4–74.6)	70.7 (59.7–79.7)	75.4 (63.4–84.4)	58.2 (53.8–62.5)
Among underweight/normal weight women (BMI ≤24.9)	62.9 (53.2–71.8)	65.1 (49.7–77.9)^§^	66.4 (48.5–80.5)^§^	53.1 (47.4–58.7)
Among overweight women (BMI 25.0–29.9)	73.6 (61.8–82.7)	—^¶^	—	65.5 (56.5–73.5)
Among obese women (BMI >30.0)	74.8 (62.0–84.4)	—	—	65.8 (54.8–75.3)
**About taking vitamins with folic acid before pregnancy^†^**				
Among women with a recent live birth	59.4 (53.0–65.5)	81.1 (71.5–88.0)	87.9 (77.3–94.0)	92.2 (89.5–94.3)
About the effects of smoking during pregnancy on a baby**				
Among women with a recent live birth	79.1 (73.4–83.9)	65.3 (54.4–74.9)	58.0 (45.3–69.7)^§^	48.3 (43.9–52.7)
Among women who reported any level of smoking in the 3 mos before pregnancy	83.9 (72.3–91.2)	—	—	62.2 (48.4–74.4)^§^
Among women who reported not smoking in the 3 mos before pregnancy	77.0 (70.1–82.7)	62.1 (50.0–72.9)	54.9 (40.7–68.4)^§^	46.7 (42.2–51.4)
About effects of drinking alcohol during pregnancy on a baby**				
Among women with a recent live birth	81.1 (75.5–85.6)	64.5 (53.4–74.2)	62.5 (49.8–73.6)^§^	50.2 (45.8–54.6)
Among women who reported drinking one or more alcoholic drinks per week on average in the 3 mos before pregnancy	86.2 (72.8–93.5)	—	—	48.6 (41.6–55.6)
Among women who reported drinking less than one alcoholic drink per week on average in the 3 mos before pregnancy	79.9 (73.6–85.1)	63.6 (51.3–74.3)	63.1 (48.0–75.9)^§^	51.3 (45.7–56.9)

**Abbreviations:** BMI = body mass index calculated as weight (kg)/(height [m])^2^, using self-reported height and weight; CI = confidence interval; FPL = federal poverty level.

* Data from eight states (Hawaii, Maine, Maryland, Michigan, Minnesota, New Jersey, Utah, and West Virginia).

^†^ Data from six states (Maryland, Michigan, Nebraska, New Jersey, Tennessee, and Vermont).

^§^ Estimate might not be reliable and should be used with caution; represents 30–59 respondents.

^¶^ Does not meet reliability standards.

** Data from five states (Maryland, Michigan, Nebraska, New Jersey, and Tennessee).

**TABLE 8:** Percentage of women with a recent live birth receiving health care services before pregnancy, by insurance coverage during the month before pregnancy **—** Pregnancy Risk Assessment Monitoring System, selected states, 2011–2012

Type of service	Had health insurance coverage	Did not have health insurance coverage
	% (95% CI)	% (95% CI)
Reported talking with a doctor, nurse, or other health care worker about how to improve her health before pregnancy*		
Among women with a recent live birth	37.6 (36.2–39.1)	14.5 (12.6–16.6)
**Reported talking with a doctor, nurse, or other health care worker before pregnancy**		
**About achieving a healthy weight before pregnancy^†^**		
Among women with a recent live birth	62.6 (59.8–65.4)	63.0 (52.7–72.2)
Among underweight/normal weight women (BMI ≤24.9)	57.6 (53.8–61.4)	64.7 (49.8–77.1)^§^
Among overweight women (BMI 25.0–29.9)	67.1 (61.4–72.3)	—^¶^
Among obese women (BMI >30.0)	71.4 (65.2–76.8)	—
**About taking vitamins with folic acid before pregnancy^†^**		
Among women with a recent live birth	83.2 (81.0–85.2)	64.5 (55.0–73.1)
About the effects of smoking during pregnancy on a baby**		
Among women with a recent live birth	58.1 (55.3–60.9)	55.6 (45.6–65.2)
Among women who reported any level of smoking in the 3 mos before pregnancy	73.3 (66.9–78.9)	—
Among women who reported not smoking in the 3 mos before pregnancy	54.6 (51.5–57.7)	57.2 (45.8–67.9)
About the effects of drinking alcohol during pregnancy on a baby**		
Among women with a recent live birth	60.2 (57.4–62.9)	58.8 (48.7–68.2)
Among women who reported drinking one or more alcoholic drinks per week on average in the 3 mos before pregnancy	54.1 (48.9–59.1)	—
Among women who reported drinking less than one alcoholic drink per week on average in the 3 mos before pregnancy	62.9 (59.6–66.1)	61.5 (50.7–71.2)

**Abbreviations:** BMI = body mass index calculated as weight (kg)/(height [m])^2^, using self-reported height and weight; CI = confidence interval.

* Data from eight states (Hawaii, Maine, Maryland, Michigan, Minnesota, New Jersey, Utah, and West Virginia).

^†^ Data from six states (Maryland, Michigan, Nebraska, New Jersey, Tennessee, and Vermont).

^§^ Estimate might not be reliable and should be used with caution; represents 30–59 respondents.

^¶^ Does not meet reliability standards.

** Data from five states (Maryland, Michigan, Nebraska, New Jersey, and Tennessee).

Of selected preconception counseling topics reported in selected states, the most frequently discussed was taking vitamins with folic acid before pregnancy (81.2%), followed by achieving a healthy weight before pregnancy (62.9%) and how drinking alcohol (60.3%) or smoking (58.2%) during pregnancy can affect a baby (Table 5). By race/ethnicity, a higher percentage of Hispanic and black women compared with white women talked with a health care professional about the effects of smoking (75.1%, 74.6%, and 50.3%, respectively) and drinking alcohol (78.0%, 75.5%, and 52.4%, respectively) during pregnancy (Table 6). By family income, the percentage of women who talked with a health care professional about taking vitamins with folic acid before pregnancy was highest among women at >400% FPL (92.2%) and lowest among women at ≤138% FPL (59.4%); conversely, a higher percentage of women with family income ≤138% FPL compared with >400% FPL spoke with a health care professional about the effects of smoking (79.1% versus 48.3%) and drinking alcohol (81.1% versus 50.2%) during pregnancy (Table 7).

#### Counseling and Screening Services Among All Women of Reproductive Age: Blood Pressure, Diabetes, Diet, and Smoking

 For women aged 18–44 years, irrespective of pregnancy status, among those without a history of hypertension (i.e., had never been told they had high blood pressure), 80.9% had their blood pressure checked by a health care professional in the past year. Among those with no previous diagnosis of diabetes who had ever been told they had high blood pressure,^§§§^ 54.5% were tested for high blood sugar or diabetes in the past year. Among women with obesity, 44.9% had a health care professional talk with them about their diet in the past year; among current smokers, 55.2% had a health care professional talk with them about smoking (Table 9). Estimates of the receipt of these services are also stratified by age (Table 9) and by race/ethnicity (Table 10).

**TABLE 9:** Percentage of women aged 18–44 years receiving preconception health and related reproductive health care services, by age **—** National Health Interview Survey, 2013

Type of service	Total (18–44 yrs)	18–19 yrs	20–24 yrs	25–34 yrs	35–44 yrs
	% (95% CI)	% (95% CI)	% (95% CI)	% (95% CI)	% (95% CI)
**Blood pressure checked by a health care professional in the past yr**					
Among women aged 18–44 yrs who had never been told they had high blood pressure	**80.9 (79.7–82.1)**	76.3 (69.6–81.9)	77.5 (74.2–80.5)	82.0 (80.2–83.6)	82.9 (81.1–84.5)
**Tested for high blood sugar or diabetes in the past yr**					
Among women aged 18–44 yrs with no previous diagnosis of diabetes who had ever been told they had high blood pressure*	**54.5 (49.7–59.2)**	—^†^	45.9 (28.1–64.9)	50.0 (41.7–58.3)	59.0 (52.9–64.9)
**Had a health care professional talk with them about their diet in the past yr**					
Among women aged 18–44 yrs	**25.9 (24.7–27.2)**	19.2 (14.4–25.0)	22.8 (20.0–25.7)	26.1 (24.2–28.1)	28.8 (26.7–31.0)
Among underweight women (BMI <18.5)	**17.1 (12.0–23.8)**	—	14.9 (7.4–27.9)^§^	22.9 (14.1–35.0)	9.4 (3.5–22.8)^§^
Among normal weight women (BMI 18.5–24.9)	**16.8 (15.2–18.6)**	13.8 (8.8–20.9)	18.6 (14.9–23.1)	15.8 (13.6–18.2)	17.6 (14.8–20.7)
Among overweight women (BMI 25.0–29.9)	**22.8 (20.5–25.3)**	12.2 (5.8–24.0)^§^	17.7 (12.7–24.3)	25.0 (21.2–29.2)	24.7 (21.1–28.7)
Among obese women (BMI >30.0)	**44.9 (42.2–47.7)**	38.2 (23.5–55.5)	40.1 (33.3–47.3)	45.5 (41.1–49.9)	47.2 (43.2–51.2)
**Had a health care professional talk with them about smoking in the past yr**					
Among current smokers aged 18–44 yrs^¶^	**55.2 (51.5–58.8)**	54.6 (34.1–73.6)	52.3 (42.9–61.6)	55.6 (50.6–60.5)	56.4 (50.7–62.0)
**Received a tetanus vaccine in the past 10 yrs**					
Among women aged 18–44 yrs	**63.3 (61.9–64.7)**	74.4 (68.2–79.7)	63.0 (59.3–66.5)	63.7 (61.6–65.8)	61.0 (58.7–63.1)
**Received an influenza vaccine in the past yr**					
Among women aged 18–44 yrs	**31.7 (30.4–33.1)**	25.1 (19.6–31.4)	24.1 (21.2–27.3)	33.0 (31.1–35.0)	35.9 (33.8–38.1)
**Received a Pap test in the past 3 yrs**					
Among women, age specific	81.6 (80.3–82.8)**	38.6 (33.4–44.0)^††^	74.6 (71.0–77.9)^§§^	83.8 (82.0–85.4)	82.5 (80.8–84.2)
**Received a mammogram in the past 2 yrs**					
Among women aged 30–44 yrs	**N/A**	N/A	N/A	16.7 (15.1–18.4)^¶¶^	53.7 (50.4–56.9)***

**Abbreviations:** BMI = body mass index calculated as weight (kg)/(height [m])^2^, using self-reported height and weight; CI = confidence interval; N/A: not applicable; Pap = Papanicolaou.

* Women had to have been told on two or more different visits that they had hypertension or high blood pressure to be classified as ever told they had high blood pressure.

^†^ Does not meet reliability standards.

^§^ Estimate might not be reliable and should be used with caution; the relative standard error (RSE) is >30% but does not exceed 50%.

^¶^ Women who reported smoking ≥100 cigarettes during their lifetime and, at the time of interview, reported smoking every day or some days.

** Because Pap tests are not recommended for women aged <21 years, this estimate only includes women aged 21–44 years.

^††^ Because Pap tests are not recommended for women <21 years, this estimate includes women aged 18–20 years to evaluate overscreening among this age group.

^§§^ Because Pap tests are not recommended for women <21 years, this estimate only includes women aged 21–24 years.

^¶¶^ Because QFP does not routinely recommend mammograms for women aged <50 years, this estimate is provided to evaluate overscreening among this age group. Because the National Health Interview Survey does not ask this question to women aged <30 years, this estimate includes women aged 30–39 years.

*** Because mammograms are not routinely recommended for women aged <40 years, this estimate only includes women aged 40–44 years.

**TABLE 10:** Percentage of women aged 18–44 years receiving preconception and related reproductive health care services, by race/ethnicity — National Health Interview Survey, 2013

Type of service	Non-Hispanic white	Non-Hispanic black	Hispanic	Non-Hispanic other or multiple races
	% (95% CI)	% (95% CI)	% (95% CI)	% (95% CI)
**Blood pressure checked by a health care professional in the past yr**				
Among women aged 18–44 yrs who had never been told they had high blood pressure	84.9 (83.3–86.4)	82.5 (79.6–85.2)	69.4 (66.5–72.1)	78.8 (74.9–82.3)
**Tested for high blood sugar or diabetes in the past yr**				
Among women aged 18–44 yrs with no previous diagnosis of diabetes who had ever been told they had high blood pressure*	55.2 (48.1–62.2)	54.7 (46.6–62.6)	62.4 (51.7–71.9)	33.1 (18.4–52.2)
**Had a health care professional talk with them about their diet in the past yr**				
Among women aged 18–44 yrs	25.6 (24.0–27.4)	34.1 (30.8–37.5)	23.8 (21.4–26.2)	20.3 (17.0–23.9)
Among underweight women (BMI <18.5)	18.8 (11.9–28.5)	—^†^	12.4 (5.0–27.7)^§^	—
Among normal weight women (BMI 18.5–24.9)	17.4 (15.3–19.8)	19.1 (14.4–25.0)	16.1 (13.0–19.9)	12.7 (9.3–17.2)
Among overweight women (BMI 25.0–29.9)	22.3 (19.0–25.9)	25.9 (20.6–32.0)	18.0 (14.5–22.2)	35.1 (25.5–46.1)
Among obese women (BMI >30.0)	46.4 (42.4–50.5)	51.3 (45.5–57.1)	38.8 (33.8–44.1)	32.4 (23.8–42.5)
**Had a health care professional talk with them about smoking in the past yr**				
Among current smokers aged 18–44 yrs^¶^	58.4 (53.9–62.8)	48.7 (40.7–56.8)	45.2 (36.0–54.8)	42.2 (29.5–56.1)
**Received a tetanus vaccine in the past 10 yrs**				
Among women aged 18–44 yrs	69.4 (67.4–71.2)	54.9 (51.4–58.3)	54.2 (51.4–57.0)	56.7 (51.9–61.4)
**Received an influenza vaccine in the past yr**				
Among women aged 18–44 yrs	32.8 (30.9–34.7)	28.0 (24.8–31.3)	27.6 (25.1–30.2)	39.9 (35.7–44.4)
**Received a Pap test in the past 3 yrs**				
Among women aged 21–44 yrs**	84.1 (82.4–85.6)	85.7 (82.8–88.2)	77.1 (74.4–79.6)	69.5 (64.7–73.9)
**Received a mammogram in the past 2 yrs**				
Among women aged 40–44 yrs^††^	55.3 (50.9–59.7)	50.0 (41.0–59.1)	49.8 (43.3–56.3)	55.7 (45.0–65.9)

**Abbreviations:** BMI = body mass index calculated as weight (kg)/(height [m])^2^, using self-reported height and weight; CI = confidence interval; Pap = Papanicolaou.

* Women had to have been told on two or more different visits that they had hypertension or high blood pressure to be classified as ever told they had high blood pressure.

^†^ Does not meet reliability standards.

^§^ Estimate might not be reliable and should be used with caution; the relative standard error (RSE) is >30% but does not exceed 50%.

^¶^ Women who reported smoking ≥100 cigarettes during their lifetime and, at the time of interview, reported smoking every day or some days.

** This estimate only includes women aged 21–44 years because Pap tests are not recommended for women aged <21 years.

^††^ This estimate only includes women aged 40–44 years because mammograms are not routinely recommended for women aged <40 years.

Receipt of preconception care and related preventive health services tended to increase with higher family income and greater continuity of insurance coverage. For each of the following, the percentage of women receiving services in the past year was higher among those with family income >400% FPL compared with ≤138% FPL and among those with continuous insurance coverage compared with no coverage over the past year: blood pressure checks by a health care professional (89.4% versus 72.9% and 87.3% versus 51.6%, respectively, for family income and insurance); testing for high blood sugar or diabetes (66.3% versus 44.1% and 61.0% versus 28.7%, respectively, for family income and insurance); and having a health care professional talk with them about their diet, among those with obesity (57.7% versus 37.4% and 49.6% versus 26.0%, respectively, for family income and insurance) (Tables 11 and 12). Among women who were current smokers, a higher percentage with continuous coverage in the past year (64.7%) or coverage with gaps (52.2%) compared with no coverage (32.1%) talked with a health care professional about their smoking (Table 12). By income, CIs overlapped across all categories, although talking with a health care professional about smoking was lowest among women with family income ≤138% FPL (50.3%), intermediate among women with family income 139%–249% FPL (55.5%) and 250%–400% FPL (57.0%), and highest among women with family income >400% FPL (64.4%) (Table 11).

**TABLE 11:** Percentage of women aged 18–44 years receiving preconception and related reproductive health care services, by family income **—** National Health Interview Survey, 2013

Type of service	≤138% FPL	139%–250% FPL	251%–400% FPL	>400% FPL
	% (95% CI)	% (95% CI)	% (95% CI)	% (95% CI)
**Blood pressure checked by a health care professional in the past yr**				
Among women aged 18–44 yrs who had never been told they had high blood pressure	72.9 (70.5–75.3)	77.3 (74.3–80.1)	83.0 (80.1–85.5)	89.4 (87.4–91.2)
**Tested for high blood sugar or diabetes in the past yr**				
Among women aged 18–44 yrs with no previous diagnosis of diabetes who had ever been told they had high blood pressure*	44.1 (37.0–51.6)	54.1 (44.6–63.4)	61.3 (49.5–71.9)	66.3 (53.2–77.3)
**Had a health care professional talk with them about their diet in the past yr**				
Among women aged 18–44 yrs	22.9 (20.8–25.0)	25.6 (22.5–29.0)	26.6 (23.8–29.6)	28.6 (26.0–31.4)
Among underweight women (BMI <18.5)	20.4 (12.2–32.1)	12.3 (5.2–26.2)^†^	10.8 (4.0–25.9)^†^	21.0 (10.1–38.4)^†^
Among normal weight women (BMI 18.5–24.9)	13.9 (11.4–16.9)	17.3 (13.3–22.2)	16.1 (12.8–20.1)	18.7 (15.9–21.9)
Among overweight women (BMI 25.0–29.9)	17.4 (13.8–21.7)	19.7 (15.0–25.5)	24.1 (19.2–29.9)	29.8 (24.6–35.7)
Among obese women (BMI >30.0)	37.4 (33.2–41.7)	41.4 (35.6–47.4)	49.7 (42.8–56.5)	57.7 (50.9–64.1)
**Had a health care professional talk with them about smoking in the past yr**				
Among current smokers aged 18–44 yrs^§^	50.3 (45.2–55.5)	55.5 (47.5–63.2)	57.0 (48.0–65.6)	64.4 (54.6–73.1)
**Received a tetanus vaccine in the past 10 yrs**				
Among women aged 18–44 yrs	56.0 (53.5–58.5)	59.7 (56.4–62.9)	64.9 (61.7–67.9)	71.8 (69.2–74.3)
**Received an influenza vaccine in the past yr**				
Among women aged 18–44 yrs	24.2 (22.2–26.4)	28.6 (25.8–31.6)	31.0 (28.2–34.0)	41.8 (38.9–44.7)
**Received a Pap test in the past 3 yrs**				
Among women aged 21–44 yrs^¶^	74.6 (72.1–76.9)	80.2 (77.2–82.8)	83.5 (80.7–86.0)	87.3 (85.2–89.2)
**Received a mammogram in the past 2 yrs**				
Among women aged 40–44 yrs**	41.2 (34.5–48.3)	49.8 (41.8–57.8)	52.4 (45.0–59.7)	63.9 (58.0–69.5)

**Abbreviations:** BMI = body mass index calculated as weight (kg)/(height [m])^2^, using self-reported height and weight; CI = confidence interval; FPL = federal poverty level; Pap = Papanicolaou.

* Women had to have been told on two or more different visits that they had hypertension or high blood pressure to be classified as ever told they had high blood pressure.

^†^ Estimate might not be reliable and should be used with caution; the relative standard error (RSE) is >30% but does not exceed 50%.

^§^ Women who reported smoking ≥100 cigarettes during their lifetime and, at the time of interview, reported smoking every day or some days.

^¶^ This estimate only includes women ages 21–44 years because Pap tests are not recommended for women aged <21 years.

** This estimate only includes women aged 40–44 years because mammograms are not routinely recommended for women aged <40 years.

**TABLE 12:** Percentage of women aged 18–44 years receiving preconception and related reproductive health care services, by continuity of health insurance coverage — National Health Interview Survey, 2013

Type of service	Had insurance coverage continuously during the past yr	Had insurance coverage with gaps during the past yr	Did not have any insurance coverage during the past yr
	% (95% CI)	% (95% CI)	% (95% CI)
**Blood pressure checked by a health care professional in the past yr**			
Among women aged 18–44 yrs who had never been told they had high blood pressure	87.3 (86.0–88.5)	81.0 (77.4–84.1)	51.6 (47.7–55.4)
**Tested for high blood sugar or diabetes in the past yr**			
Among women aged 18–44 yrs with no previous diagnosis of diabetes who had ever been told they had high blood pressure*	61.0 (55.2–66.5)	45.7 (34.2–57.7)	28.7 (19.0–40.9)
**Had a health care professional talk with them about their diet in the past yr**			
Among women aged 18–44 yrs	28.5 (27.0–30.0)	26.0 (22.6–29.7)	14.1 (11.7–16.8)
Among underweight women (BMI <18.5)	18.8 (12.2–27.7)	26.7 (12.6–48.0)^†^	—^§^
Among normal weight women (BMI 18.5–24.9)	18.9 (16.9–21.0)	12.0 (8.7–16.3)	8.8 (5.9–12.9)
Among overweight women (BMI 25.0–29.9)	26.2 (23.3–29.4)	22.1 (16.1–29.6)	10.4 (7.0–15.3)
Among obese women (BMI >30.0)	49.6 (46.3–52.9)	43.2 (36.3–50.3)	26.0 (20.1–33.0)
**Had a health care professional talk with them about smoking in the past yr**			
Among current smokers aged 18–44 yrs^¶^	64.7 (60.1–69.0)	52.2 (44.1–60.2)	32.1 (25.5–39.6)
**Received a tetanus vaccine in the past 10 yrs**			
Among women aged 18–44 yrs	67.2 (65.5–68.8)	62.5 (58.5–66.4)	46.6 (43.0–50.2)
**Received an influenza vaccine in the past yr**			
Among women aged 18–44 yrs	37.0 (35.4–38.6)	24.0 (20.8–27.6)	14.1 (11.8–16.7)
**Received a Pap test in the past 3 yrs**			
Among women aged 21–44 yrs**	86.4 (85.1–87.6)	81.8 (78.3–84.8)	60.5 (56.7–64.1)
**Received a mammogram in the past 2 yrs**			
Among women aged 40–44 yrs^††^	59.4 (55.6–63.0)	47.0 (37.2–57.1)	26.8 (18.4–37.2)

**Abbreviations:** BMI = body mass index calculated as weight (kg)/(height [m])^2^, using self-reported height and weight; CI = confidence interval; Pap = Papanicolaou.

* Women had to have been told on two or more different visits that they had hypertension or high blood pressure to be classified as ever told they had high blood pressure.

^†^ Estimate might not be reliable and should be used with caution; the relative standard error (RSE) is >30% but does not exceed 50%.

^§^ Does not meet reliability standards.

^¶^ Women who reported smoking ≥100 cigarettes during their lifetime and, at the time of interview, reported smoking every day or some days.

** This estimate only includes women ages 21–44 years because Pap tests are not recommended for women aged <21 years.

^††^ This estimate only includes women aged 40–44 years because mammograms are not routinely recommended for women aged <40 years.

####  Vaccinations Among All Women of Reproductive Age

 Receipt of vaccines according to recommendations varied across the different vaccine types. Approximately one third (31.7%) of women had received an influenza vaccination in the past year and approximately two thirds (63.3%) had received a tetanus vaccine in the past 10 years (Table 9). Receipt of an influenza vaccine in the past year increased across age groups and was lower among younger than among older women (25.1% and 24.1% for women aged 18–19 and 20–24 years, respectively, versus 35.9% among women aged 35–44 years). Conversely, receipt of a tetanus vaccine in the past 10 years was higher among women aged 18–19 years (74.4%) compared with those in older age groups (range: 61.0%–63.7%).

Receipt of recommended vaccinations tended to rise across increasing income categories and with continuity of insurance coverage. By income, the percentage receiving a tetanus vaccine in the past 10 years and an influenza vaccine in the past year was higher among women with family income >400% FPL (71.8% and 41.8%, respectively, for tetanus and influenza) compared with those with family income ≤138% FPL (56.0% and 24.2%, respectively, for tetanus and influenza) and 139%–250% FPL (59.7% and 28.6%, respectively, for tetanus and influenza) (Table 11). Similarly, by insurance coverage, receipt of tetanus and influenza vaccines was lower among women who had no insurance in the past year (46.6% and 14.1%, respectively, for tetanus and influenza) compared with women who had continuous insurance coverage (67.2% and 37.0%, respectively, for tetanus and influenza) or coverage with gaps (62.5% and 24.0%, respectively, for tetanus and influenza) (Table 12).

#### Related Preventive Health Services: Pap Tests and Mammograms

 Among women aged 21–44 years, 81.6% received a Pap test in the past 3 years. Among women aged 40–44 years, 53.7% received a mammogram in the past 2 years (Table 9). Receipt of Pap tests and mammograms tended to increase across increasing income categories and with continuity of insurance coverage. The percentage of women aged 21–44 years who received a Pap test in the past 3 years was higher among those with family income >400% FPL (87.3%) and 251%–400% FPL (83.5%) compared with those with family income ≤138% FPL (74.6%) (Table 11). By insurance coverage, receipt of a Pap test was higher among women with continuous insurance coverage over the past year (86.4%) or coverage with gaps (81.8%) compared with those with no coverage (60.5%) (Table 12). Similarly, the percentage of women aged 40–44 years who received a mammogram in the past 2 years was higher among those with family income >400% FPL (63.9%) compared with those with family income ≤138% FPL (41.2%) and 139%–250% FPL (49.8%) (Table 11). The percentage of women aged 40–44 years who received a mammogram in the past 2 years was higher among those with continuous insurance coverage over the past year (59.4%) compared with those with no coverage (26.8%) (Table 12).

## Discussion

The findings of this report suggest that many women and men of reproductive age were not receiving recommended preventive health care services during 2011–2013. Differences in the use of preventive services occurred by age, race/ethnicity, family income, and stability of insurance coverage. However, although the pattern across strata for age and race/ethnicity varied for different services, receipt of preventive services was consistently lower among women and men with lower family income and among persons who had no health insurance coverage compared with those with continuous coverage or coverage with gaps. The 2014 publication of QFP establishes standards for selected preventive services that can be monitored for improvements from baseline levels in 2011–2013.

The findings in this report are consistent with previous findings which also suggest that not all persons of reproductive age have been receiving recommended preventive services and that many differences in the receipt of services exist by age, race/ethnicity, family income, and insurance coverage (*36*–*46*). This report is unique in providing a comprehensive baseline assessment of the full set of preventive services recommended in QFP for persons of reproductive age and can serve as an important resource for identifying opportunities to address gaps in service use. Among women of reproductive age, many report that the place where they receive family planning care is their usual source of medical care and they typically receive care in no other place (*36*,*47*). Among men of reproductive age, because of their infrequent use of preventive health services and perceived lack of need for reproductive health services, turning standalone services visits into opportunities for providing more comprehensive care might be important (*48*–*50*). As a result, having information on the range of preventive services received by persons of reproductive age available in one report can help to identify potential opportunities for integrating provision of services across settings.

Identifying gaps in use of recommended services underscores the importance of evaluating efforts to improve preventive service delivery. Because receipt of services varied with continuity of insurance coverage, ongoing surveillance would be useful to better understand the impact of changes in the U.S. health care system that influence insurance coverage. In addition, ongoing surveillance could provide information about the impact of other efforts to improve preventive health services delivery, including continued support for safety net programs for persons who are uninsured, have low incomes, or are in need of confidential services (*51*,*52*); development of payment and reimbursement strategies; integration of family planning services into larger systems of care; and development and use of clinical performance measures to monitor and promote improvements in delivery of preventive health care services (*53*–*55*). The data in this report can be used to monitor the progress of such efforts to increase the use of preventive services recommended in QFP for women and men of reproductive age. 

## Limitations

The findings in this report are subject to at least four limitations. First, although the population included in each estimate was aligned to the subpopulation recommended for receipt of services to identify gaps, for certain services, the national surveys lacked sufficient information for precise alignment (e.g., testing for STDs and HIV, which depends on local prevalence for certain subgroups). Second, national data were not available for services among women with a recent live birth; therefore, estimates for certain services were made on the basis of a limited number of states and are unlikely to be nationally representative. Third, self-reported data might be subject to recall bias. Finally, the analyses did not control for confounding factors, describe the quality of care received, or explain the reasons for receipt or nonreceipt of services. 

## Conclusion

Understanding patterns of preventive health services use among women and men of reproductive age can be an important first step toward developing research priorities, information for decision makers, and public health practice. The findings in this report can help to identify subpopulations that have not been receiving recommended services and can provide information for making decisions about resource allocation. The findings can also guide future research to identify factors that promote or serve as barriers to services use and ways to increase the use of preventive health services and improve reproductive health outcomes. Monitoring progress in adoption of the standards for the provision of quality services recommended in QFP is an important tool for improving care for women and men of reproductive age.

## Footnotes

* For purposes of this report, receipt of contraceptive services among women includes receiving a birth control method or a prescription, receiving a checkup for birth control, receiving counseling about birth control, receiving a sterilizing operation, receiving counseling about a sterilizing operation, receiving emergency contraception, or receiving counseling about emergency contraception.

† Women were considered at risk for unintended pregnancy if they had ever had vaginal intercourse; were neither pregnant, seeking pregnancy, nor postpartum; and neither they nor their partner were noncontraceptively sterile. Postpartum women were identified to have completed a recent pregnancy ≤2.5 months before interview according to the following coding specifications: https://www.cdc.gov/nchs/data/nsfg/nsfg_2011-2013_app3a_femresprecodespecs_v2.pdf.

§ Moderately effective contraceptive methods include injectable, patch, ring, pill, and diaphragm; with moderately effective methods, 6%–12% of women become pregnant during the first year of typical use. **Source:** Trussell J. Contraceptive failure in the United States. Contraception 2011;83:397–404.

¶ Less effective contraceptive methods include condoms, withdrawal, morning-after pill, foam, sponge, suppository or insert, jelly or cream, periodic abstinence, and other (unspecified) methods; with less effective methods, 18%–28% of women become pregnant during the first year of typical use. **Source:** Trussell J. Contraceptive failure in the United States. Contraception 2011;83:397–404.

** Permanent and long-acting reversible methods include male and female sterilization (e.g., vasectomy, tubal sterilization), intrauterine devices (IUDs), and contraceptive implants. With permanent and long-acting reversible methods, less than 1% of women become pregnant during the first year of typical use. **Source:** Trussell J. Contraceptive failure in the United States. Contraception 2011;83:397–404.

†† Women were considered to have infertility at the time of NSFG interview if neither they nor their current husband or partner was surgically sterile, and they had not become pregnant during the previous 12 months or longer, despite the fact they and their husband or partner were continuously married or cohabiting, they were sexually active each month, and they had not used contraception.

§§ Had more than one opposite sex partner in the past year, or had an opposite sex partner with other partners in the past year.

¶¶ Data on counseling before the most recent pregnancy on how to achieve a healthy pregnancy were from Hawaii, Maine, Maryland, Michigan, Minnesota, New Jersey, Utah, and West Virginia; data on counseling about achieving a healthy weight and taking vitamins with folic acid before pregnancy were from Maryland, Michigan, Nebraska, New Jersey, Tennessee, and Vermont; and data on the effects of smoking and drinking alcohol during pregnancy on a baby were from Maryland, Michigan, Nebraska, New Jersey, and Tennessee.

*** The relative standard error (RSE) is calculated by dividing the standard error of an estimate by the estimate itself, then multiplying by 100.

††† Medical services in addition to advice for achieving pregnancy included infertility testing, ovulation stimulation, surgery to correct blocked tubes, artificial insemination, and other types of medical help.

§§§ Women had to have been told on two or more different visits that they had hypertension or high blood pressure to be classified as ever told they had high blood pressure.

## Appendix A

### Services Recommended in *Providing Quality Family Planning Services: Recommendations of CDC and the U.S. Office of Population Affairs*

**Table:**

**Service**	**Recommendation**
**Contraceptive services**	Offer contraceptive services to all clients who wish to delay or prevent pregnancy.
**Pregnancy testing and counseling**	Offer pregnancy testing and counseling to all clients seeking this service.
**Achieving pregnancy**	Advise clients how to achieve pregnancy if they wish to become pregnant and are seeking this service.
**Basic infertility services**	Offer basic infertility services to infertile clients (i.e., those who have failed to achieve pregnancy after 12 mos or longer of regular unprotected intercourse) seeking this service. Earlier assessment may be justified for clients with identified risk factors.
**Preconception health services**	
Folic acid*^,†^	All women planning or capable of pregnancy should be counseled about the need to take a daily supplement containing folic acid.
IPV*^,†^	Screen all women of childbearing age for IPV and provide/refer to intervention services, as indicated.
Alcohol and other drug use^†^	Screen clients for use of alcohol and other drugs and provide/refer for behavior counseling, as indicated.
Tobacco use^†^	Screen clients for tobacco use and provide/refer for tobacco cessation interventions, as indicated; provide adolescents with interventions to prevent initiation of tobacco use.
Immunizations	Screen for immunization status and provide/refer for immunization, as indicated by ACIP: influenza (annually); tetanus (once every 10 yrs); and HPV and hepatitis B (one-time receipt of vaccine series).
Depression	Screen all clients for depression when staff-assisted depression care supports are in place to ensure accurate diagnosis, effective treatment, and follow-up. USPSTF notes that the optimum interval for screening for depression is unknown; however, they suggest that a pragmatic approach might be to screen all adults who have not been screened previously and to use clinical judgment in consideration of risk factors, comorbid conditions, and life events to determine whether additional screening of high-risk patients is warranted.
BMI^†^	Assess client’s height, weight, and BMI and provide/refer adults with obesity for intensive counseling and behavioral interventions.
Blood pressure	At the time QFP was published, the USPSTF recommendation was to screen persons with blood pressure <120/80 mm Hg routinely. For adults who are prehypertensive (i.e., 120–130 mm Hg/80–89 mm Hg), the Joint National Committee on Prevention, Detection, Evaluation, and Treatment of High Blood Pressure recommends annual screening. AAP recommends annual screening for adolescents. In 2015, USPSTF updated its recommendation on blood pressure screening to indicate that adults at increased risk (i.e., black, with high normal blood pressure, with obesity or overweight, or aged >40 yrs) should be screened every year, while persons at low risk (aged 18–39 yrs with no risk factors) should be screened every 3–5 yrs.
Diabetes	At the time QFP was published, the USPSTF recommendation was to screen for type 2 diabetes in asymptomatic adults with sustained blood pressure (either treated or untreated) >135/80 mm Hg. In 2015, USPSTF changed its recommendations to indicate adults aged 40–70 yrs with obesity or overweight should be screened for type 2 diabetes. USPSTF notes that evidence on the optimal rescreening interval for adults with an initial normal glucose test result is limited; however, rescreening every 3 yrs might be a reasonable approach for adults with normal blood glucose levels.
**STD services**	
Chlamydia	At the time QFP was published, the CDC recommendation was to screen annually for chlamydia infection all sexually active young females aged ≤25 yrs and among sexually active women aged >25 yrs with risk factors. Screening of male clients can be considered at sites with high prevalence of chlamydia infection and MSM. Recommendations are to provide/refer for treatment, as indicated. In 2015, CDC STD guidelines changed the age for screening all sexually active young women from ≤25 yrs to <25 yrs. Recommendations for women above this age with additional risk factors and male clients remain the same.
Gonorrhea	Screen all women at risk for gonorrhea infection annually, including women aged <25 yrs. Screen MSM clients. Provide/refer for treatment, as indicated.
Syphilis	Screen persons at risk for syphilis infection, including MSM, commercial sex workers, persons who exchange sex for drugs, persons in adult correctional facilities, and persons living in communities with high prevalence of syphilis.
HIV/AIDS	Screen all clients aged 13–64 yrs for HIV/AIDS on a routine basis and rescreen all persons at high risk for HIV infection annually. Refer for care, as indicated.
Hepatitis C	Provide one-time testing for hepatitis C infection for persons born during 1945–1965 and routine screening for persons at high risk for hepatitis C infection. Persons with HIV infection should be tested at least annually for hepatitis C infection. Provide/refer for treatment, as indicated.
**Related preventive health**	
Cervical cytology*	Screen women aged 21–65 yrs with cervical cytology (Pap smear) every 3 yrs or women aged 30–65 yrs with a combination of cytology and HPV testing every 5 yrs. Refer for further diagnosis and treatment, as indicated.
Breast cancer screening*	Screen women aged 50–74 yrs biennially with mammogram; women aged <50 yrs can be considered if other conditions support providing the service. Refer for further diagnosis and treatment, as indicated.

**Abbreviations:** AAP = American Academy of Pediatrics; ACIP = Advisory Committee on Immunization Practices; BMI = body mass index; HIV/AIDS = human immunodeficiency virus/acquired immunodeficiency syndrome; HPV = human papillomavirus; IPV = intimate partner violence; mm Hg = millimeters of mercury; MSM = men who have sex with men; Pap = Papanicolaou; QFP = *Providing Quality Family Planning Services: Recommendations of CDC and the U.S. Office of Population Affairs*; USPSTF = U.S. Preventive Services Task Force; STD = sexually transmitted disease.

* This service is only recommended for female clients.

^†^ USPSTF has not recommended a screening interval because evidence is lacking to determine the optimal interval.

## Appendix B

### Adverse Health Outcomes Targeted for Prevention Through Services Recommended in *Providing Quality Family Planning Services: Recommendations of CDC and the U.S. Office of Population Affairs*, by Age

**Table:**

**Outcome**	**Total (15–44 yrs)**		**15–19 yrs**	**20–24 yrs**	**25–29 yrs**	**30–34 yrs**	**35–39 yrs**	**40–44 yrs**
**Unintended pregnancies, 2011** ***** **^,^** **^†^**								
No. of unintended pregnancies (in 1,000s)	**2,779** **^§^**		430	878	691	444	328^¶^	
Unintended pregnancy (rate per 1,000 population)**	**45** **^§^**		41	81	66	43	16^¶^	
Pregnancies unintended (%)	**45** **^§^**		75	59	42	31	34^¶^	
**Teen births, 2015** **^††^**								
No. of teen births (in 1,000s)	**N/A**		230	N/A	N/A	N/A	N/A	N/A
Teen births (rate per 1,000 population)^§§^	**N/A**		22.3	N/A	N/A	N/A	N/A	N/A
Teen births that are repeat births (%)^¶¶^	**N/A**		16.7	N/A	N/A	N/A	N/A	N/A
**Birth spacing, 2014** *******								
Singleton births conceived <18 mos after previous live birth (%)	**28.9** **^†††^**		22.4^§§§^	27.1	29.3	34.9	44.1^¶^	
**Infant health, 2015** **^††^**								
Births that are preterm (%)^¶¶¶^	**9.6** **^†††^**		9.9^§§§^	9.3	8.9	9.4	11.1	13.7
Births with low birth weight (%)****	**8.1** **^†††^**		9.5^§§§^	8.4	7.5	7.5	8.7	10.8
**Infertility, 2011–2013^††††, §§§§^**								
Married and cohabiting women (% [95% CI])	**6.3 (4.9–7.7)**		—**^¶¶¶¶^**	4.9 (1.3–8.5)	5.5 (2.6–8.5)	4.2 (2.4–6.0)	8.5 (4.6–12.3)	8.3 (5.5–11.1)
**Obesity, 2011–2012*******								
Overweight women (% [95% CI])**^†††††^**	**25.2 (21.4–29.1)**		14.9 (9.7–20.0)	21.7 (14.2–29.3)	29.5 (25.0–33.9)^§§§§§^		28.3 (22.4–34.2)^¶¶¶¶¶^	
Obese women (% [95% CI])**^†††††^**	**30.2 (27.1–33.4)**		16.4 (10.0–22.9)	26.9 (18.3–35.5)	34.4 (29.4–39.4)^§§§§§^		34.9 (29.9–39.9)^¶¶¶¶¶^	
**Hypertension, 2011–2012*******								
Women with high blood pressure or medication for high blood pressure (% [95% CI])******	**8.8 (6.8–10.7)**		—	—	6.4 (4.3–8.5)^§§§§§^		19.6 (15.0–24.2)^¶¶¶¶¶^	
**Diabetes, 2011–1012*******								
Women ever told by health care professional they had diabetes (% [95% CI]s	**2.5 (1.7–3.4)**		—	—	1.2 (0.6–1.8)^§§§§§^		5.9 (3.7–8.2)^¶¶¶¶¶^	
**Diagnoses of HIV infection, 2014 estimated,^††††††^ both sexes**								
No. of diagnoses of HIV infection	**32,931**		1,854	7,983	7,963	6,093	4,750	4,288
Rate of diagnoses of HIV infection (per 100,000 population)	**13.9^§§§§§§^**		8.7	34.4	35.8	28.0	23.5	20.6
**Diagnoses of HIV infection, 2014 estimated,^††††††^ males**								
No. of diagnoses of HIV infection	**27,371^¶¶¶¶¶¶^**		1,505	7,083	6,853	4,970	3,659	3,301
**Diagnoses of HIV infection, 2014 estimated,^††††††^ females**								
No. of diagnoses of HIV infection	**5,561**		349	901	1,110	1,123	1,091	987
**Diagnoses of AIDS, 2014 estimated,******* noncumulative, both sexes**								
No. of diagnoses of AIDS	**12,255**		233	1,485	2,555	2,641	2,615	2,726
Rate of diagnoses of AIDS (per 100,000 population)	**6.6^§§§§§§^**		1.1	6.4	11.5	12.1	13.0	13.1
**Diagnoses of AIDS, 2014 estimated,^¶¶¶¶¶¶^ noncumulative, males**								
No. of diagnoses of AIDS	**9,334**		166	1,236	2,090	2,013	1,878	1,951
**Diagnoses of AIDS, 2014 estimated,^¶¶¶¶¶¶^ noncumulative, females**								
No. of diagnoses of AIDS	**2,922**		68	249	465	628	737	775
**STDs, 2014, number and rate, women*********								
No. of chlamydia cases reported	**981,230**		303,294	405,876	161,793	67,060	29,545	13,662
Chlamydia cases reported per 100,000 women	**627.2** **^†††^**		2,941.0	3,651.1	1,523.4	633.7	300.9	130.3
No. of gonorrhea cases reported	**156,589**		44,399	59,329	28,899	13,988	6,654	3,320
Gonorrhea cases reported per 100,000 women	**101.3** **^†††^**		430.5	533.7	272.1	132.2	67.8	31.7
No. of syphilis cases reported	**1,654**		262	503	361	248	177	103
Syphilis cases reported per 100,000 women	**1.1** **^†††^**		2.5	4.5	3.4	2.3	1.8	1.0
**STDs, 2014, number and rate, men*********								
No. of chlamydia cases reported		**412,036**	77,908	159,804	91,729	45,990	22,894	13,711
Chlamydia cases reported per 100,000 men		**278.4^†††††††^**	718.3	1,368.3	837.0	430.6	234.0	132.3
No. of gonorrhea cases reported		**169,124**	23,981	56,714	40,602	24,349	14,129	9,349
Gonorrhea cases reported per 100,000 men		**120.1^†††††††^**	221.1	485.6	370.5	228.0	144.4	90.2
No. of syphilis cases reported		**14,277**	761	3,632	3,727	2,635	1,868	1,654
Syphilis cases reported per 100,000 men		**11.7^†††††††^**	7.0	31.1	34.0	24.7	19.1	16.0

**Abbreviations:** AIDS = acquired immunodeficiency syndrome; HIV = human immunodeficiency virus; CI = confidence interval; N/A = not applicable; STDs = sexually transmitted diseases.

* **Source:** Finer LB, Zolna MR. Declines in unintended pregnancy in the United States, 2008–2011. N Engl J Med 2016;374:843–52.

^†^ Pregnancies were classified as unintended if they were either mistimed or unwanted; pregnancies were classified as intended if they were desired at the time they occurred or sooner.

^§^ Includes women aged ≥15 years. Adolescents aged <15 years were excluded because of insufficient data.

^¶^ Includes women aged ≥35 years.

** Number of unintended pregnancies per 1,000 women aged 15–44 years or specific age group. For women aged ≥35 years, the numerator is the number of unintended pregnancies among women aged ≥35 years and the denominator is the number of women aged 35–44 years.

^††^ **Source:** Martin JA, Hamilton BE, Osterman MJK, Driscoll AK, Mathews TJ. Births: final data for 2015. Natl Vital Stat Rep 2017;66:1–69.

^§§^ Births per 1,000 female teens aged 15–19 years.

^¶¶^ The percentage of births among teenagers aged 15–19 years that were second or higher order, among all births where birth order was not missing.

*** **Source:** Thoma ME, Copen CE, Kirmeyer SE. Short interpregnancy intervals in 2014: differences by maternal demographic characteristics. NCHS Data Brief 2016;240:1–8.

^†††^ Includes women of all ages.

^§§§^ Includes all women aged ≤19 years.

^¶¶¶^ Less than 37 completed weeks of gestation on the basis of the obstetric estimate.

**** Birth weight <2,500 grams.

^††††^ **Source:** National Survey of Family Growth, 2011–2013; special data tabulation.

^§§§§^ Percentage of all married and cohabiting women aged 15–44 years who are infertile (i.e., who are not surgically sterile and have had at least 12 consecutive months of unprotected sexual intercourse without becoming pregnant).

^¶¶¶¶^ Does not meet reliability standards.

***** **Source:** National Health and Nutrition Examination Survey, 2011–2012; special data tabulation.

^†††††^ For women aged 15–19 years, overweight was defined as ≥85th and <95th percentile of body mass index (BMI) for age; obesity was defined as ≥95th percentile. For women aged 20–44 years, overweight was defined as BMI ≥25 and <30; obesity was defined as BMI ≥30.

^§§§§§^ Includes women aged 25–34 years.

^¶¶¶¶¶^ Includes women aged 35–44 years.

****** For women aged 15–19 years, hypertension was defined as ≥95th percentile systolic or diastolic blood pressure given age and height percentile or use of medication to control blood pressure. For women aged 20–44 years, hypertension was defined as ≥140 mm Hg systolic blood pressure, ≥90 mm Hg diastolic blood pressure, or use of medication to control blood pressure.

^††††††^ **Source:** National HIV Surveillance System. Data include persons with a diagnosis of HIV infection regardless of stage of disease at diagnosis. The estimated numbers and rates of diagnoses of HIV infection were made on the basis of data from 50 states and six dependent areas. Estimated numbers resulted from statistical adjustment that accounted for reporting delays and missing risk-factor information, but not for incomplete reporting. CDC. HIV surveillance report, 2014; vol. 26. https://www.cdc.gov/hiv/pdf/library/reports/surveillance/cdc-hiv-surveillance-report-2014-vol-26.pdf (Tables 1b and 5b).

^§§§§§§^ Includes women and men of all ages.

^¶¶¶¶¶¶^ **Source:** National HIV Surveillance System. The estimated numbers and rates of stage 3 infection (AIDS) were made on the basis of data from 50 states and six dependent areas. Estimated numbers resulted from statistical adjustment that accounted for reporting delays and missing risk-factor information, but not for incomplete reporting. CDC. HIV Surveillance Report, 2014; vol. 26. https://www.cdc.gov/hiv/pdf/library/reports/surveillance/cdc-hiv-surveillance-report-2014-vol-26.pdf (Tables 2b and 6b).

******* **Source:** Nationally Notifiable Disease Surveillance System. 2014 sexually transmitted disease surveillance report. Chlamydia: https://www.cdc.gov/std/stats14/tables/10.htm; gonorrhea: https://www.cdc.gov/std/stats14/tables/21.htm; and syphilis: https://www.cdc.gov/std/stats14/tables/35.htm.

^†††††††^ Includes men of all ages.
